# The TFAMoplex—Conversion of the Mitochondrial Transcription Factor A into a DNA Transfection Agent

**DOI:** 10.1002/advs.202104987

**Published:** 2022-01-17

**Authors:** Michael Burger, Seraina Kaelin, Jean‐Christophe Leroux

**Affiliations:** ^1^ Swiss Federal Institute of Technology Zurich (ETHZ) Department of Chemistry and Applied Biosciences Institute of Pharmaceutical Sciences Vladimir‐Prelog‐Weg 3 Zurich 8093 Switzerland

**Keywords:** DNA nanoparticles, non‐viral gene delivery, protein engineering, protein‐based DNA carrier

## Abstract

Non‐viral gene delivery agents, such as cationic lipids, polymers, and peptides, mainly rely on charge‐based and hydrophobic interactions for the condensation of DNA molecules into nanoparticles. The human protein mitochondrial transcription factor A (TFAM), on the other hand, has evolved to form nanoparticles with DNA through highly specific protein‐protein and protein‐DNA interactions. Here, the properties of TFAM are repurposed to create a DNA transfection agent by means of protein engineering. TFAM is covalently fused to *Listeria monocytogenes* phospholipase C (PLC), an enzyme that lyses lipid membranes under acidic conditions, to enable endosomal escape and human vaccinia‐related kinase 1 (VRK1), which is intended to protect the DNA from cytoplasmic defense mechanisms. The TFAM/DNA complexes (TFAMoplexes) are stabilized by cysteine point mutations introduced rationally in the TFAM homodimerization site, resulting in particles, which show maximal activity when formed in 80% serum and transfect HeLa cells in vitro after 30 min of incubation under challenging cell culture conditions. The herein developed TFAM‐based DNA scaffolds combine interesting characteristics in an easy‐to‐use system and can be readily expanded with further protein factors. This makes the TFAMoplex a promising tool in protein‐based gene delivery.

## Graphical abstract

The protein human mitochondrial transcription factor A (TFAM) binds and condenses DNA into nanoparticles. TFAM is used as a scaffold to form so called TFAMoplexes for DNA transfection of mammalian cells. The TFAM protein is coupled to a membranolytic phospholipase and a kinase to overcome important hurdles in the transfection process.

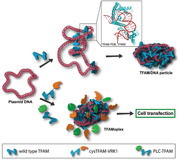

## 1 Introduction

Despite the progress in gene therapy research and the availability of a few gene therapy products, the safe and efficient delivery of DNA remains challenging. The most commonly used nucleic acid delivery systems are viruses and synthetic particles made from various materials such as lipids or polymers.^[^ ^1^ ^]^ While viruses are highly efficient delivery vehicles, they pose several threats, have limited packaging capacity, and cannot easily be modified without losing efficacy. The assembly of non‐viral systems, on the other hand, is often based solely on electrostatic or hydrophobic interactions (e.g., ionizable lipids or dendrimers). In the presence of serum proteins, such particles are prone to destabilization and aggregation. Further, these systems interact poorly with the cellular machinery, for example specific cell surface receptors or the cellular transport machineries. This usually translates into low transfection efficiencies.^[^ ^2^ ^]^

Here we made use of the human mitochondrial transcription factor A (TFAM) to deliver DNA into cells. In contrast to classical transfection reagents such as lipofectamine or polyethyleneimine, TFAM's ability to bind double‐stranded DNA with high affinity and in a sequence‐independent manner makes it a unique candidate for delivering DNA. Unlike supercharged proteins or cationic peptides, which have been used to aggregate plasmids into particles,^[^ ^3^, ^4^ ^]^ TFAM is capable of organizing single DNA molecules into compact, nucleosome‐like structures, both within mitochondria and in cell‐free media.^[^ ^5^ ^]^ Importantly, these structures can form spontaneously without helper proteins or chaperones, which is in contrast to other protein‐based DNA‐compacting machineries, such as multicomponent nucleosomes in nuclear chromatin.^[^ ^6^ ^]^

**Figure 1:**
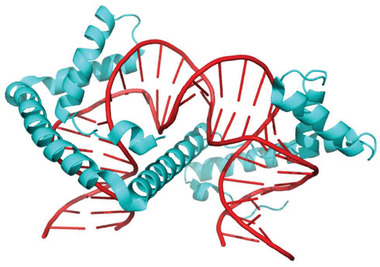
Crystal structure of a TFAM monomer (cyan) bound to double‐stranded DNA (red). Figure generated with Pymol using PDB file 3TMM.^[^ ^13^ ^]^

Endogenous TFAM is expressed in the cytoplasm and transported to the mitochondria, where it constitutes the most frequent protein component and plays a role in the compaction and regulation of the mitochondrial genome.^[^ ^7^ ^]^ TFAM contains two DNA‐binding high mobility group (HMG)‐box domains, which are connected by an alpha helix (**Figure** **1**). Both termini are unstructured and point toward opposite sides of the protein. When bound to DNA, TFAM homodimerizes via an antiparallel interaction of its helix 3.^[^ ^8^ ^]^ At low protein concentrations, TFAM dimerization results in the formation of local protein/DNA condensates.^[^ ^7^, ^9^ ^]^ When the concentration is increased, the DNA is fully clustered and nanocomplexes consisting of TFAM and a single DNA molecule are formed. This DNA‐compacting mechanism of TFAM constitutes an important advantage over other HMG‐box proteins that were previously used as transfection agents. The nuclear protein HMGB1, for example, exhibits high affinity for DNA, but conclusive evidence (i.e., electron microscopy studies) for the spontaneous formation of compact particles with DNA, either within the cell or in vitro, is missing.^[^ ^10^ ^]^ Although studies have shown that the weak DNA compaction ability of HMGB1 can be compensated with the addition of poly(ethyleneimine) or cationic peptides,^[^ ^11^, ^12^ ^]^ a system that does not require additional components, such as TFAM, would be more desirable. To the best of our knowledge, TFAM has not yet been considered as a DNA transfection agent.

To equip TFAM with additional functionalities, it can be linked to other proteins by genetic fusion. An important step following the cellular uptake of DNA is its escape from the endolysosomal compartment. To endow TFAM/DNA nanoparticles with the ability to exit the endosome, we incorporated the phosphatidylcholine‐specific phospholipase C (pc‐PLC, abbreviated here as PLC) from *Listeria monocytogenes (L. m.)* ^[^ ^14^ ^]^ into the TFAM/DNA nanoparticles. PLC hydrolyzes a broad range of phospholipids with a preference for phosphatidylcholine,^[^ ^15^ ^]^ which is the most abundant lipid in the inner leaflet of endosomal membranes.^[^ ^16^ ^]^ The enzyme is most active at pH 5, a condition that is prevalent in the maturating endosomes and is almost inactive at physiological pH.^[^ ^17^ ^]^ While *L. m*. PLC was never used for non‐viral gene delivery systems, phospholipases originating from insect venoms have been applied for this purpose before.^[^ ^18^, ^19^ ^]^

Once inside the cytoplasm, the DNA cargo must be transported toward and into the nucleus for transcription. This process is hindered by several hurdles, such as the crowded nature of the cytosol and the nuclear pore, a highly regulated barrier that only allows selected substrates to pass through. A hurdle that might significantly impact the cytoplasmic mobility of DNA is the presence of a powerful DNA defense system dependent on the protein barrier‐to‐autointegration factor (BAF).^[^ ^20^ ^]^ BAF binds cytoplasmic DNA, clusters it, and attracts endoplasmic reticulum membranes via its interaction with LEM (LAP2, emerin, MAN1)‐domain proteins. It can be inhibited by the human VRK1, which is responsible for BAF inactivation by hyperphosphorylation prior to mitotic nuclear envelope disassembly.^[^ ^20^, ^21^, ^22^, ^23^, ^24^, ^25^, ^26^ ^]^

In this work, we developed recombinant fusion constructs of TFAM with *L. m*. PLC or human VRK1 to form DNA/protein complexes (TFAMoplexes) that effectively transfect HeLa cells. By rational insertion of cysteine mutations in the TFAM dimerization site, the particles could be further stabilized to remain functional in serum, resulting in the successful transfection of HeLa cells after only 30 min of incubation with a low plasmid dose.

## 2 Results

### 2.1 Incorporation of PLC

**Figure 2:**
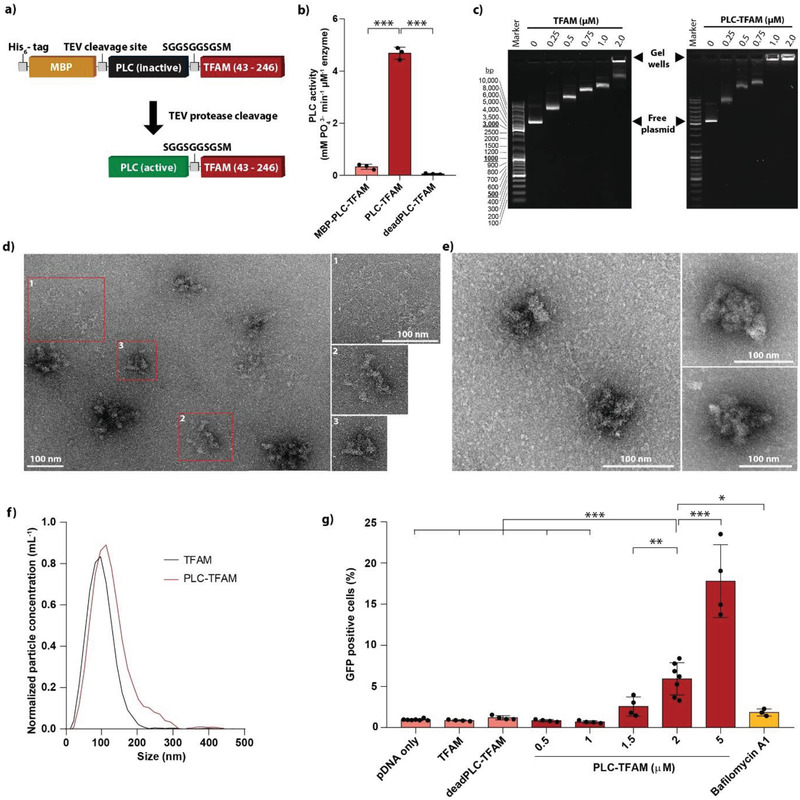
The PLC‐TFAM construct. a) Schematic representation of the PLC‐TFAM construct before and after TEV protease cleavage. Tags and linker sequences are depicted with grey squares, the MBP protein in orange, PLC in black (inactive form) and green (active form), and the TFAM moiety (amino acids 43–246) in red. b) PLC activity measurements by modified malachite green assay. The enzymatic activity of PLC‐TFAM was compared to the construct MBP‐PLC‐TFAM and deadPLC‐TFAM. Shown is the release of PO_4_^3–^ per minute of incubation with 1 × 10^–6^ m PLC construct. Each dot represents the mean of an independent triplicate experiment. Mean ± SD (*N* = 3). c) Gel mobility shift assay of the enhanced green fluorescent protein plasmid (pEGFP) with increasing concentrations of either wild‐type TFAM or PLC‐TFAM. The positions of the free plasmid band and the gel wells are indicated by black arrowheads. TEM images of typical pEGFP DNA complexes with d) 1 × 10^–6^ m TFAM or e) 1 × 10^–6^ m PLC‐TFAM. f) Representative NTA measurements of particle hydrodynamic diameter distributions. pEGFP DNA was complexed with either wild‐type TFAM (2 × 10^–6^ m, black curve) or PLC‐TFAM (1 × 10^–6^ m, red curve). The data were normalized with respect to the peak values. g) Flow cytometry analysis of HeLa cells transfected with pEGFP/PLC‐TFAM particles for 30 min in 10% fetal bovine serum (FBS). Negative controls consisted of DNA particles formed with 5 × 10^–6^ m wild‐type TFAM or deadPLC‐TFAM. Bafilomycin A1 (500 × 10^–9^ m, yellow bar) was used as an inhibitor of endosomal acidification with 2 × 10^–6^ m plc‐tfam. Two hundred ng pDNA per mL growth medium was used. Each dot represents the mean of an independent triplicate experiment. Mean ± SD (N = 3‐7), *p < 0.05, **p < 0.01, ***p < 0.001.

We hypothesized that genetically fusing TFAM to PLC might enable the complexes to escape the endosome and deliver its bound DNA to the cytosol. The fusion construct composed of *L. m*. PLC and truncated human TFAM lacking its N‐terminal mitochondrial transport signal was termed PLC‐TFAM (**Figure** **2**a). It was further extended with an N‐terminal his_6_‐MBP moiety, which served as a solubility and purification tag on one hand, but also as an artificial pro‐domain that inhibits PLC activity during bacterial expression. Upon cleavage with tobacco etch virus (TEV) protease, the his_6_‐MBP tag was removed to generate the wild‐type PLC N‐terminus, which is then buried in the protein structure to constitute a part of the enzyme's active site.^[^ ^27^ ^]^ PLC activity was measured on 1‐palmitoyl‐2‐oleoyl‐*sn*‐glycero‐3‐phosphocholine (POPC) liposomes with a modified malachite green assay^[^ ^28^ ^]^ (Figure 2b), and compared to an inactive phospholipase construct (deadPLC‐TFAM) harboring a tryptophan to glycine mutation on active site residue 52. As expected, both MBP‐PLC‐TFAM and deadPLC‐TFAM did not hydrolyze POPC to a significant extent, while the TEV‐digested PLC‐TFAM construct was active. In the presence of plasmid DNA (pDNA), the PLC‐TFAM protein retained 50% of its PLC activity compared to the free protein (Figure S1, Supporting Information).

The DNA‐binding function of wild‐type TFAM (amino acids 43‐246) and PLC‐TFAM was tested in a gel mobility shift assay with pDNA (Figure 2c). The observed mobility shift patterns were similar for both systems and in accordance with reports from literature.^[^ ^29^ ^]^ However, while wild‐type TFAM/DNA complexes migrated into the gel up to a protein concentration of 1 × 10^–6^ m, for PLC‐TFAM, DNA retention in the gel wells occurred at lower protein concentrations. This suggests that the N‐terminal fusion of PLC affected the physiochemical properties of the complexes but did not prevent TFAM/DNA binding.

The particles were visualized by transmission electron microscopy (TEM) imaging (Figure 2d,e), and hydrodynamic diameters were determined by nanoparticle tracking analysis (NTA) (Figure 2f). As reported previously,^[^ ^30^ ^]^ at moderate concentrations (e.g., 1 × 10^–6^ m) wild‐type TFAM yielded populations with different degrees of compaction, ranging from partially open plasmids (Figure 2d, inset 1) to fully condensed 100‐nm particles (Figure 2d, inset 3). This trend was also observed with the PLC‐TFAM fusion construct (Figure 2e and Figure S2, Supporting Information), which clearly showed uncomplexed DNA loops protruding from the particles at low TFAM concentrations, but not at higher concentrations. The zeta potential of the PLC‐TFAM/DNA complexes formed with 1 × 10^–6^ m protein and 10 ng µL^­–1^ DNA was −24.5 ± 1.8 mV.

Next, we evaluated the complexes’ ability to transport a reporter DNA plasmid into HeLa cells. Particles were prepared with an enhanced green fluorescent protein (EGFP)‐encoding plasmid (pEGFP) and incubated with the cells for 30 min in growth medium supplemented with 10% fetal bovine serum (FBS). Particles that were formed in the presence of more than 2 × 10^–6^ m PLC‐TFAM resulted in significant GFP expression (Figure 2g). Up to 20% of the cells were transfected when the particles were generated with 5 × 10^–6^ m fusion construct PLC‐TFAM. On the other hand, DNA complexed with 5 × 10^–6^ m wild‐type TFAM or with the inactive phospholipase construct, did not produce any detectable EGFP expression. When the transfection was performed with 2 × 10^–6^ m plc‐tfam in the presence of 500 × 10^–9^ m bafilomycin A1, which is an inhibitor of endosomal acidification, the transfection efficiency decreased significantly. This indicates, that endolysosomal acidification is necessary for successful transfection and supports our hypothesis that the phospholipase activity promotes the endosomal escape of the complexes.

### 2.2 Incorporation of VRK1

**Figure 3:**
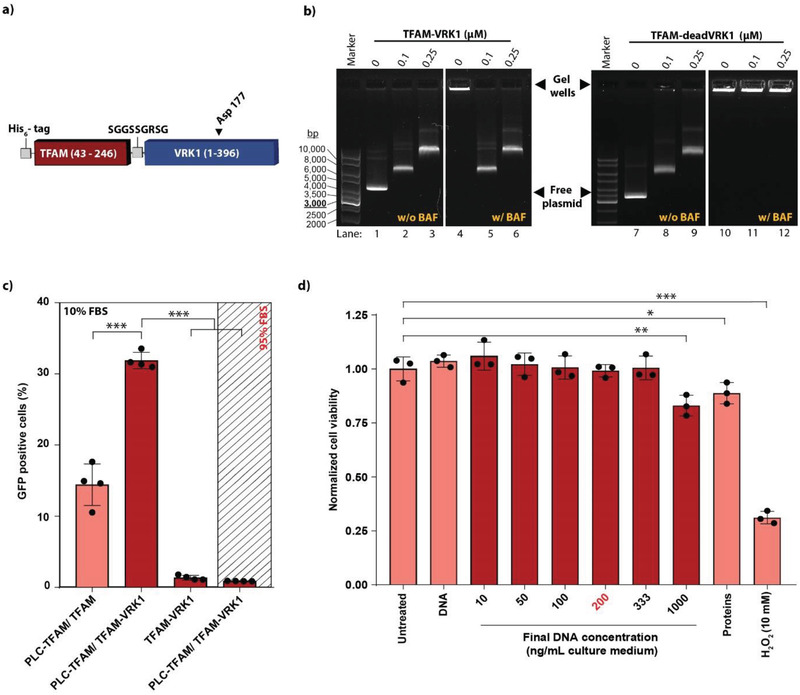
The TFAM‐VRK1 constructs. a) Schematic representation of the TFAM‐VRK1 construct with a N‐terminal his_6_‐tag, TFAM (amino acids 43–246, red), a short flexible linker, and VRK1 (amino acids 1–396, blue). The mutated aspartic acid at position 177 in the deadVRK1 protein is indicated with a black arrowhead. b) Gel mobility shift assay in the presence (w/) and absence (w/o) of BAF to test TFAM and VRK1 activities within the fusion construct. In lanes 1–3 and 7–9 pDNA was incubated with increasing TFAM‐VRK1 or TFAM‐deadVRK1 concentrations, respectively, in the absence of BAF. In lanes 4–6 and 10–12, 1 × 10^–6^ m BAF was included. In lanes 4 and 10–12, the DNA was retained in the gel wells by BAF. In lanes 5 and 6, VRK1 inactivated BAF and restored the TFAM‐VRK1‐specific mobility shift pattern. The positions of the free plasmid band and the gel wells are indicated by the black arrowheads. c) Flow cytometry analysis of HeLa cells transfected with various DNA/TFAM particles (200 ng pDNA per mL growth medium). The total TFAM concentration during particle formation was 2 × 10^–6^ m. The transfections were performed in medium supplemented with either 10% FBS or 95% FBS (white or dashed box, respectively). Each dot represents the mean of an independent triplicate experiment. Mean ± SD (*N* = 4). d) Cell viability assay. HeLa cells were incubated for 17 h with the DNA particles formed with TFAM‐VRK1 and PLC‐TFAM in an equimolar ratio in growth medium supplemented with 10% FBS. The final DNA concentration in the medium is indicated. In the “proteins” control, only the proteins were added to the cells without DNA (same concentration as in the 1000 ng ml^–1^ DNA sample). The DNA concentration which is used in a standard 30 min transfection experiment is highlighted in red. Hydrogen peroxide was used as a positive control for cytotoxicity. Data were normalized to the untreated cell control. Each dot represents the mean of an independent triplicate experiment. Mean ± SD (*N* = 3), *p < 0.05, **p < 0.01, ***p < 0.001.

To protect the DNA in the cytoplasm from BAF‐dependent retention, the carrier system was expanded by a second factor, human BAF kinase VRK1 (**Figure** **3**a). As a negative control for VRK1 activity, a TFAM‐deadVRK1 construct with a kinase‐inactivating mutation (D177A) was produced. First, we demonstrated that the VRK1 constructs complexed DNA and protected it from interacting with BAF in a gel mobility shift assay (Figure 3b). For phosphorylation to occur, the assay was performed in T4 ligase buffer containing adenosine triphosphate (ATP). TFAM‐VRK1 generated the same characteristic DNA band shift pattern (lanes 2, 3, 8, 9) as observed before, indicating that the TFAM moiety within the fusion construct was forming complexes with the plasmids. In the presence of BAF, unprotected plasmid DNA (without TFAM protein) was clustered and retained in the gel wells (lanes 4, 10). Similarly, DNA that was complexed with TFAM‐deadVRK1 could not enter the gel in the presence of BAF (lanes 11, 12). TFAM‐VRK1, on the other hand, prevented BAF‐induced DNA clustering and produced a similar band shift pattern as observed in the absence of BAF (lanes 5, 6). These data show that TFAM‐VRK1 is able to inhibit BAF‐induced DNA clustering in vitro.

The particles formed with pDNA and TFAM‐VRK1 were visualized by TEM imaging (Figure S3a, Supporting Information). Their mean diameter measured by NTA was 120 nm (Figure S3b, Supporting Information) and the zeta potential −15.3 ± 1.1, which was significantly higher than the potential obtained from PLC‐TFAM/DNA complexes.

Next, we wanted to test if we could combine the activities of PLC‐TFAM and TFAM‐VRK1 to achieve higher transfection efficiencies. Indeed, the TFAM‐VRK1 protein significantly increased the percentage of transfected HeLa cells when particle formation was performed in a 1:1 molar ratio (PLC‐TFAM:TFAM‐VRK1, Figure 3c), while the zeta potential of these complexes was −14.2 ± 3.8 mV. Interestingly, the improved transfection efficiency was not due to kinase activity, as we will eventually demonstrate under the final transfection conditions in paragraph 2.6. We then tested the impact of the particles containing both PLC‐TFAM and TFAM‐VRK1 on HeLa cell viability (Figure 3d). After 17 h of incubation with escalating DNA doses, we observed that a significant decrease in cell viability only occurred at DNA concentrations of 1 µg mL^–1^ cell medium or higher. Standard transfection experiments, however, were performed with 30 min of incubation and 200 ng mL^–1^ DNA, in which no cytotoxicity was observed.

To develop an effective transfection agent, we tested the PLC‐TFAM construct under more challenging conditions. In addition to the small DNA doses and short incubation times, we increased the serum levels during particle incubation with cells to 95% mimicking a more biologically relevant environment. Notably, under these conditions the transfection efficiency of the constructs containing wild‐type TFAM dropped to basal levels (Figure 3c).

### 2.3 Particle Stabilization

**Figure 4:**
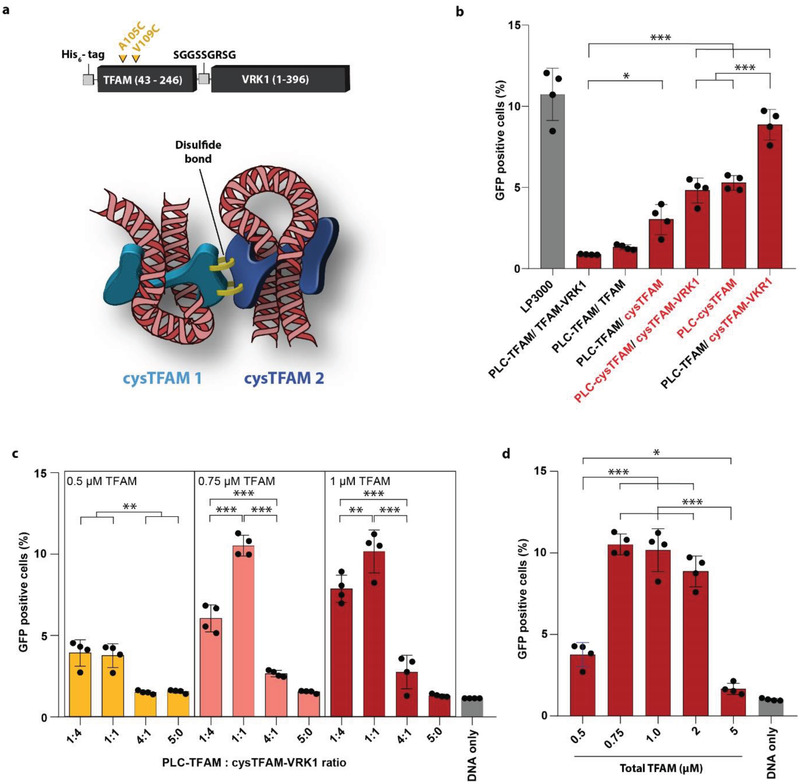
The double cysteine mutant cysTFAM. a) Schematic representation of the intended TFAM homodimer stabilization by disulfide bridge formation (yellow clamps). DNA is shown in red, TFAM proteins in turquoise and blue. b) Flow cytometry analysis of HeLa cells transfected with the DNA/TFAM complexes or with lipofectamine 3000 (LP3000) in 95% FBS. Two hundred ng pDNA per mL culture medium was used. The DNA/TFAM particles were formed with various TFAM variants in a 1:1 molar ratio and a total TFAM concentration of 1 × 10^–6^ m. Shown are the percentages of GFP positive cells. c,d) Flow cytometry analysis of HeLa cells 24 h after transfection with the DNA/TFAM complexes in 95% FBS. The cells were incubated with 200 ng pDNA per mL culture medium for 30 min. In (c) the pDNA/TFAM particles were formed with increasing total concentrations of TFAM and the indicated PLC‐TFAM/cysTFAM‐VRK1 ratios. In (d) pDNA/TFAM particles were formed with the indicated total TFAM concentrations at an equimolar ratio of PLC‐TFAM and cysTFAM‐VRK1. The bars shown for TFAM concentrations of 0.5, 0.75, and 1 × 10^–6^ m are taken from the data of panel c. Each dot represents the mean of an independent triplicate experiment. Mean ± SD (*N* = 4), *p < 0.05, **p < 0.01, ***p < 0.001.

To increase the stability of the DNA/TFAM particles in high serum conditions two cysteine mutations were introduced in alpha helix 3 of TFAM, which is required for homodimerization.^[^ ^8^ ^]^ We speculated that the introduced cysteines would form inter‐molecular disulfide bridges and, therefore, protect the DNA/TFAM particles from decondensing in the oxidizing environment of the extracellular space. In the cytoplasm, on the other hand, the disulfide bridges should be cleaved by the prevalent reducing conditions. The cysteines replaced the outward facing residues alanine 105 and valine 109 (**Figure** **4**a). The resulting TFAM double mutant (i.e., TFAM A105C, V109C) was named cysTFAM and was used to generate the constructs cysTFAM, cysTFAM‐VRK1 and PLC‐cysTFAM. In combination with plasmid DNA cysTFAM‐VRK1 formed particles with similar dimensions as wild‐type TFAM or PLC‐TFAM (Figure S4, Supporting Information). Also, a 1:1 mixture of cysTFAM‐VRK1 and PLC‐TFAM resulted in the formation of particles with plasmid DNA. This indicated that the double cysteine mutant of TFAM behaved similarly in particle formation as unmodified TFAM. Further, the cysTFAM‐VRK1/PLC‐TFAM/DNA particles were shown to be non‐toxic to HeLa cells at the applied concentrations with prolonged incubation times (Figure S5, Supporting Information).

Indeed, all the tested double cysteine mutant versions of TFAM significantly increased the transfection efficiency after 30 min of incubation in high levels of serum when combined with PLC‐TFAM or its double cysteine mutant version PLC‐cysTFAM (Figure 4b). The particles obtained with cysTFAM and PLC‐TFAM transfected 3% of the cells. This value increased to 5% when using the PLC‐cysTFAM construct, either alone or in combination with cysTFAM‐VRK1. Surprisingly, the highest transfection efficiency was not achieved with a mixture of cysTFAM‐VRK1 and PLC‐cysTFAM, but with an equimolar combination of PLC‐TFAM (no additional cysteines) and cysTFAM‐VRK1, yielding 9% GFP positive cells. Similar transfection efficiencies were observed with the positive control Lipofectamine 3000 (LP3000) under identical conditions. The mean GFP fluorescence intensities obtained in these experiments are shown in Figure S6, Supporting Information.

We then varied both the total TFAM concentration and the ratio of the two proteins during particle formation (Figure 4c). Generally, the efficiencies decreased when PLC‐TFAM was applied in excess over the cysTFAM‐VRK1 construct. Maximal transfection efficiencies of 11% (both regarding the percentage of transfected cells as well as GFP intensity) were achieved at a total TFAM concentration of 0.75 × 10^–6^ m in a 1:1 molar ratio. Notably, when the total TFAM concentration during particle formation was increased to more than 2 µm, the transfection efficiency decreased and reached basal levels at 5 × 10^–6^ m total TFAM (Figure 4d). We assume that high concentrations of the TFAM cysteine mutants do not allow the ordered condensation of the DNA and, therefore, the formation of nanoparticles. Accordingly, in the subsequent experiments, a total TFAM concentration of 0.75 × 10^–6^ m and a 1:1 molar ratio (VRK1‐cysTFAM : PLC‐TFAM) were used for particle formation. Complexes prepared in this way are hereinafter referred to as “TFAMoplex.”

The impact of the incubation time on the transfection efficacy of the TFAMoplex is shown in Figure S7, Supporting Information. The percentage of GFP expressing cells increased to 10% during the first 60 min of incubation. No further increase was observed for longer incubation times. LP3000, on the other hand, performed similarly at short incubation times (≤30 min) but outperformed the TFAMoplex after prolonged incubation.

From these experiments, we can conclude that the inserted cysteines greatly improved the performance of the system in serum. In the sections below, we will demonstrate that the cysteine modified TFAMoplexes not only retained their potency in high serum transfection conditions, but even allowed to form the complexes directly in serum. Further investigations on the presence of disulfide bridges among the TFAM proteins is presented in paragraph 2.7 applying the final transfection conditions.

### 2.4 Inverted Setup Transfection

**Figure 5:**
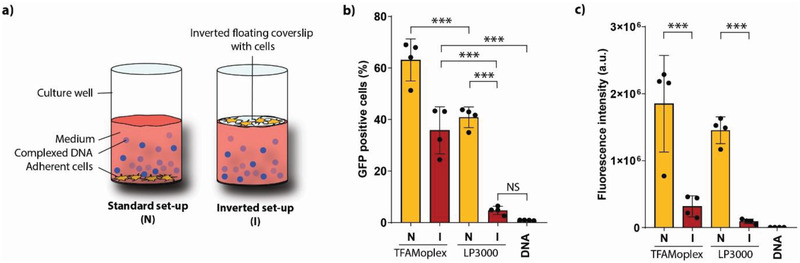
a) Schematic representation of the normal and inverted transfection setup. In the latter, the cells are adhered to a floating inverted coverslip on top of the medium containing the DNA complexes (blue spheres). Flow cytometry analysis of HeLa cells transfected for 2 h in 10% FBS with a pEGFP plasmid in the normal or inverted setup with either the TFAMoplexes or LP3000. Shown are the percentage of b) GFP positive cells and c) the GFP fluorescence intensity. Two hundred ng pDNA mL^–1^ culture medium was applied. Each dot represents the mean of an independent triplicate experiment. Mean ± SD (*N* = 4), ***p < 0.001.

Most non‐viral DNA transfection agents, including Lipofectamine, are known to depend on sedimentation effects to achieve sufficient DNA uptake in a cell culture setting. We investigated if this was also the case for the TFAMoplexes by transfecting HeLa cells both on the bottom of a multiwell plate (normal setup), and in an inverted setting on a floating plastic coverslip (inverted setup) (**Figure** **5**a). Following a 2‐h incubation in 10% serum conditions, the percentage of GFP positive cells decreased from 63% in the normal, to 36% in the inverted setup (Figure 5b). This was significantly better compared to LP3000, where the percentage dropped from 41% to 5%, respectively. While the TFAMoplexes transfected a comparably large percentage of cells in the inverted setup, the fluorescence intensity was weaker compared to cells transfected at the bottom of the well (Figure 5c). Again, this difference was even more pronounced in the case of LP3000.

### 2.5 TFAMoplex Formation in FBS

**Figure 6:**
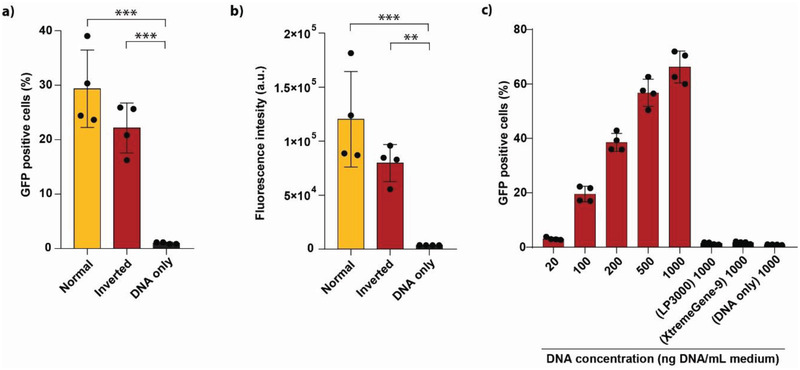
TFAMoplex formation in 80% FBS. The particles were formed in FBS and applied on HeLa cells in 99% FBS for 30 min. The EGFP expression was analyzed by flow cytometry. Two hundred ng pDNA per mL culture medium (FBS) was used and the transfections were performed in the normal and inverted setting. Shown is a) the percentage of GFP positive cells and b) the fluorescence intensities. c) TFAMoplex dose escalation. The complexes were formed in 80% FBS and applied on HeLa cells in 99% FBS for 30 min with the indicated DNA concentrations. LP3000 and XtremeGene‐9 were used at the highest DNA concentration in comparison. Each dot represents the mean of an independent triplicate experiment. Mean ± SD (N=4). **p < 0.01, ***p < 0.001.

To challenge the system even further, we tested the formation of the TFAMoplexes (i.e., cysTFAM‐VRK1, PLC‐TFAM and pDNA) directly in serum instead of PBS. The complexes were prepared in 80% FBS. Then they were added to the cells which were kept in pure FBS so that the final serum concentration in the transfection medium was ≈99%. The transfections were conducted in the normal and inverted setting (**Figure** **6**). To our surprise, the serum formed TFAMoplexes were active in a 30‐min transfection experiment, with 28% GFP‐positive cells after treatment in the normal setup (Figure 6a,b). In the inverted setup the transfection efficiency decreased to 20%. Lipoplex‐based transfection systems, such as LP3000 and XtremeGene‐9, on the other hand, did not efficiently transfect HeLa cells when particle formation was performed in 80% FBS. We escalated the TFAMoplex dose under these harsh conditions (i.e., TFAMoplex formation in 80% FBS, 30 min transfection in 99% FBS) (Figure 6c). With a pDNA concentration of 1 µg per mL of cell culture medium a transfection efficiency of 66% was achieved. The transfection efficiencies of the TFAMoplexes (i.e., cysTFAM‐VRK1 and PLC‐TFAM with pEGFP) and LP3000 are summarized in Table S1, Supporting Information.

### 2.6 Contribution of VRK1 Kinase to TFAMoplex Performance

As demonstrated in paragraph 2.2., the incorporation of VRK1 into the TFAMoplexes significantly increased the transfection efficiency of the system. Here, we investigated whether the increase was indeed due to kinase activity.^[^ ^31^ ^]^ Therefore, we formed the TFAMoplexes with the “kinase dead” construct cysTFAM‐deadVRK1, and compared the transfection efficiency with the complexes containing the active kinase. As above, the complexes were formed in 80% serum with equimolar ratios of both proteins and transfection was conducted for 30 min. Interestingly, both systems resulted in similar percentages of transfected cells, indicating that the kinase activity was not contributing to the performance of the TFAMoplex (Figure S8, Supporting Information). The improved potency was also not due to the potential interaction of VRK1 with the nuclear import machinery.^[^ ^31^ ^]^ When VRK1 in cysTFAM‐VRK1 was replaced with a nuclear localization signal (NLS), the potency of the system dropped significantly (Figure S9, Supporting Information). These experiments indicate, that VRK1 is improving the transfection efficiency based on a property or mechanism which cannot be currently rationalized.

### 2.7 Contribution of TFAM Cysteine Mutations to TFAMoplex Performance

According to our design plan, we expected to see disulfide crosslinking between the cysTFAM proteins. However, when we analyzed the DNA/cysTFAM‐VRK1 particles by non‐reducing gel electrophoresis, disulfide bridge‐coupled proteins were not observed (Figure S10, Supporting Information). We then investigated whether both cysteine mutations in TFAM were required for transfection in serum. We created two control constructs containing a single cysteine mutation A105C‐TFAM‐VRK1 and V109C‐TFAM‐VRK1. When these constructs were used to form TFAMoplexes with PLC‐TFAM and plasmid DNA, the transfection efficiency was similar to the standard TFAMoplex containing the double cysteine mutant (Figure S11, Supporting Information). We then cloned a further control construct (serTFAM‐VRK1), in which residues A105 and V109 were both mutated into serines (no thiols). If active in serum, this construct would eventually allow to rule out the formation of disulfide bridges in the TFAM dimerization site since serine is incapable of such interactions. Indeed, TFAMoplexes which were formed in serum containing serTFAM‐VKR1 were able to transfect HeLa cells in FBS with good efficiency (16% versus 22% for serTFAM‐VRK1 and cysTFAM‐VRK1, respectively).

### 2.8 Particle Uptake

**Figure 7:**
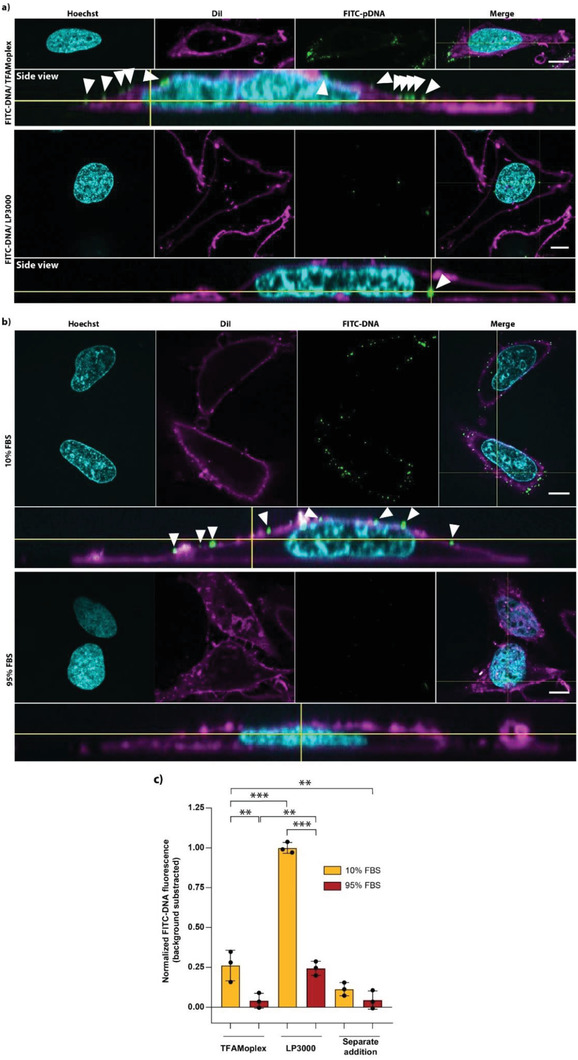
DNA uptake by HeLa cells. a) Representative confocal images of HeLa cells 2 h after incubation with FITC‐DNA containing TFAMoplex (top) or LP3000 (bottom) in growth medium supplemented with 10% FBS. Shown are single z‐slices in the different channels with the “side view” of the composite image. In the side view subfigures, the location of the FITC‐DNA foci is indicated with white arrowheads. The yellow crosshair indicates the “cutting edge” respective to the composite image and serves orientation. Cyan: Hoechst DNA staining. Magenta: DiI membrane staining. Green: FITC‐pDNA. b) FITC‐DNA and the TFAMoplex proteins were added to the cells separately. The experiment was performed in medium supplemented with 10% or 95% FBS. The cells were fixed and analyzed after 1 h. Scale bars: 10 µm. c) Flow cytometry analysis of HeLa cells incubated for 1 h with FITC‐DNA (200 ng mL^–1^ cell culture medium) using TFAMoplex, LP3000, or the separate addition of TFAMoplex proteins with naked FITC‐DNA. The experiment was performed in growth medium supplemented with 10% FBS (yellow bars) and 95% FBS (red bars). The data were normalized with respect to the mean LP3000 values in 10% FBS and the background signal (cells incubated with FITC‐DNA only) was subtracted. Each dot represents the mean of an independent triplicate experiment. Mean ± SD (*N* = 3), **p < 0.01, ***p < 0.001.

In order to analyze the uptake of the complexed DNA by HeLa cells in the normal set‐up, the TFAMoplexes were prepared with fluorescein (FITC) labeled plasmid DNA and visualized by laser confocal microscopy (**Figure** **7**a and Figure S12, Supporting Information). When the transfection was performed in 10% FBS, the FITC signal appeared as distinct spots on the plasma membrane (stained with DiI dye, magenta). Even intensive washing of the cells after transfection with acidic or heparan sulfate containing buffers did not result in the removal of the fluorescent DNA from the cell surface (Figure S13, Supporting Information). On the other hand, cells that were incubated with the lipoplexes did not retain such high DNA concentrations on the cell surface, and normally just showed few bright DNA clusters in the cytoplasmic region.

The confocal laser microscopy study was repeated, this time adding the FITC‐DNA and the proteins separately to the cell growth medium (Figure 7b). After 1 h of incubation, the cells were fixed and analyzed. Clearly, the FITC spots on the cell surface also formed under these conditions in growth medium with 10% FBS. In 95% FBS, the signal was very weak and did not generally result in cell surface staining. Also, the cytoplasmic region was mostly free of FITC‐DNA clusters shortly after transfection. However, since fluoresceine is quenched at acidic pH, plasmids present in the late endosome or lysosomes might not give a signal. This could result in an underestimation of DNA internalization.

FITC‐DNA association with HeLa cells was quantified by flow cytometry after 1 h of incubation with the TFAMoplex or LP3000 in medium with 10% or 95% serum (Figure 7c). Under these conditions, naked FITC‐DNA was not taken up by the cells in measurable quantities. In 10% FBS, the cells transfected with the TFAMoplex showed 27% of the FITC signal intensity obtained with LP3000. In high serum levels, the fluorescence intensity decreased to 4% (respective to LP3000 positive control in 10% FBS) with the TFAMoplex system and to 26% with LP3000. When the DNA and proteins were added separately to the cell medium, we still measured a significant FITC‐DNA signal in 10% FBS, while the signal in 95% FBS was very weak, as expected from the confocal studies (Figure 7b).

## 3 Discussion

Mammalian cells developed multiple defense mechanisms to protect their genome from exogenous nucleic acids. An efficient DNA carrier should be able to overcome all the barriers and control the fate of its DNA cargo until the moment of nuclear transcription or genome integration. This could be achieved by co‐delivering the DNA with highly specialized supporter proteins, each addressing another hurdle in a safe and efficient manner. But first, the DNA must be compacted by a suitable scaffold to form nanoparticles that are stable in serum and can then be expanded by additional factors.

TFAM is an interesting protein scaffold, since it binds and condenses DNA in a sequence non‐specific way, is abundantly found in all our mitochondria, can easily be produced from bacteria, and has free termini, where fusion factors can be attached.^[^ ^13^ ^]^ We demonstrated that in the fusion constructs, the activity of the different protein moieties (i.e., TFAM, PLC, VRK1) was retained and that TFAM, even with large fusion adducts, formed particles in the 100 nm size range.

A potential problem of using TFAM as a transfection agent, could be its role in innate immunity, both inside the cell as an enhancer of the cGAS‐STING pathway, and in the extracellular space.^[^ ^32^, ^33^, ^34^ ^]^ However, the protein itself is not eliciting an immune response, but rather augments the reaction against oxidized mitochondrial DNA or GC‐rich sequences of bacterial origin.^[^ ^35^ ^]^ Further, the adverse effects of TFAM in innate immunity were shown to occur at low TFAM concentrations by the introduction of characteristic DNA U‐turns. At higher TFAM concentrations, when the DNA is fully compacted, the activation of the cGAS/STING pathway can even be inhibited.^[^ ^32^ ^]^

The transfection experiments were generally performed with low amounts of DNA (200 ng mL^­–1^ growth medium), in the presence of 10–99% serum, and with short incubation times. These conditions were selected to minimize sedimentation effects as well as other artifacts that often lead to an overestimation of the performance of any given transfection agent in a cell culture setting and, therefore, limited reproducibility in vivo.

We found that PLC‐TFAM is necessary and sufficient to achieve transfection of HeLa cells. However, we must consider that DNA particle formation is performed with excess TFAM and that protein/DNA complex formation underlies constant binding and detachment of the single TFAM units. Therefore, it is unclear yet to which extent free protein contributes to the measured effect, and how active the different moieties remain within the DNA/protein complex regarding their folding state and substrate accessibility. Also, when we assembled the TFAMoplexes in 80% serum (instead of PBS), their transfection potency was retained or even improved, provided that the cysteine or serine mutant versions of TFAM were involved. This indicates that TFAM/DNA particle formation occurs not only in a protein‐free buffer, but also in the presence of serum proteins. This property highlights the full potential of specialized protein‐based systems, and clearly distinguishes the TFAMoplex from other transfection agents that mostly rely on charge‐based interactions. The ability to organize DNA with a human protein in a complex environment, such as in serum, could provide new prospects to the field of gene delivery.

By adding a second construct, TFAM‐VRK1, in an equimolar ratio the transfection efficiency was significantly increased. Currently, we cannot explain the contribution of VRK1 on the transfection process. We can exclude kinase activity as the driving factor since the kinase dead construct performs equally well in transfection experiments. VRK1 contains an endogenous nuclear localization signal and interacts with the Ran GTPase.^[^ ^31^ ^]^ This could have potentially promoted nuclear uptake and explained the altered transfection behavior. However, when cysTFAM‐VRK1 was replaced with cysTFAM‐NLS, containing a c‐terminal nuclear localization signal, the transfection efficiency of the system decreased to almost basal levels. Another explanation could be found in the physicochemical properties of the TFAM‐VRK1 construct, which contains a net positive charge of +26 and has a molecular weight of 72 kDa, while PLC‐TFAM only bears +16 charges and has a molecular weight of 53 kDa. The sizes of the PLC‐TFAM/DNA complexes and the PLC‐TFAM/cysTFAM‐VRK1/DNA complexes were similar, but the zeta potential of the latter was less negative, potentially impacting cell uptake. This change in surface properties, but also possible internal structural rearrangements, could eventually affect any step in the transfection process. The differences in DNA complex formation among the TFAM fusion proteins were also supported by gel mobility shift assays, where DNA complexed with TFAM‐VRK1 or PLC‐TFAM was retained in the gel wells already at lower protein concentrations (0.5 × 10^–6^ m) than with wild‐type TFAM (>2 × 10^–6^ m).

The question remains whether active VRK1 incorporated into the particles can protect the DNA from BAF within the cell. After entering the cytoplasm, some of the DNA is quickly clustered and retained by the BAF system. This manifests in cytoplasmic DNA foci that appear with many different transfection methods (e.g., lipofection, electroporation, microinjection).^[^ ^36^, ^37^ ^]^ In the present work, the impact of BAF cluster formation during TFAMoplex‐based transfection was not investigated. However, the presented data indicate, that the intracellular transport of the DNA might be more efficient with the TFAMoplex compared to LP3000. This speculation is based on three findings: first, both systems showed similar transfection efficiencies. Second, significantly less DNA was associated to the HeLa cells after TFAMoplex transfection than with LP3000. And third, with our system most of the DNA‐associated fluorescence seemed to be confined to the cell surface, while it was localized in the cytoplasm with LP3000.

The stabilization of nanocarriers with disulfide bridges is often applied on polymeric, lipid, and peptide‐based systems and is a well‐established tool for the covalent but reversible crosslinking of particle subunits.^[^ ^38^, ^39^ ^]^ In our case, the insertion of cysteines in a potential TFAM‐TFAM homodimerization site did not result in the formation of disulfide bridges. However, the additional cysteines (wild‐type TFAM contains 2 cysteines in its termini) had a remarkable effect on TFAMoplex formation and transfection in serum. Interestingly, the TFAMoplexes were most potent, if they were composed of 50% normal TFAM and 50% cysTFAM proteins. Also, the insertion of just a single cysteine mutation in position 105 or 109, or the insertion of two serines, allowed serum transfection. Currently, we do not understand the cause of these observations and can only speculate, that the modified residues alter the TFAM‐TFAM interaction in a favorable manner. A deeper understanding, however, of the protein interactions within the complexes will certainly allow us to further improve the performance of the TFAMoplex.

Finally, we identified cellular uptake of the DNA/TFAM particles as the major bottleneck in the transfection process. After only few minutes of incubation, the particles stick to the cellular surface, where they seem to remain for several hours. Even by extensive washing with acidic buffers or heparan sulfate the particles could not be removed from the surface. This was not the case for lipoplex based transfection or in the presence of high serum levels. Currently, it is unclear why the adsorbed particles do not seem to be endocytosed more efficiently.

## 4 Conclusion

We developed protein fusion constructs containing the highly specialized DNA‐compacting factor TFAM, which was extended with either the membranolytic activity of PLC or the BAF inhibiting kinase VRK1. In combination, these proteins spontaneously formed ≈100‐nm sized particles with plasmid DNA, and efficiently transfected HeLa cells in challenging cell culture settings. The addition of other enzymes or protein domains to the TFAM termini is comparably simple and could, as the system is further developed, allow the TFAMoplexes to interact with the cellular machinery in every stage of the transfection pathway. The incorporation of two cysteine residues in the TFAM homodimerization site strongly improved the performance of the system in high serum levels and enabled particle formation in the presence of serum. This highlights the advantages of this protein system over purely charge‐based complexing agents and could give rise to new gene delivery approaches. A major shortcoming of the TFAMoplex, which is shared with other transfection systems, is its limited uptake into the target cells. This problem must be addressed to eventually use the system for non‐cancer cell types and, eventually, in vivo. Also, the cellular fate of the TFAMoplexes requires further investigation, such as the impact of BAF on cytoplasmic mobility and nuclear uptake. Overall, TFAM is a versatile scaffold and a promising new tool for non‐viral gene delivery.

## 5 Experimental Section

### Plasmids

pET His6 TEV LIC cloning vector (1B) was a gift from Scott Gradia (Addgene plasmid # 29653, Addgene, Cambridge, MA). Human_TFAM_NoMTS_pET28 was a gift from David Chan (Addgene plasmid # 34705). pDONR223‐VRK1 was a gift from William Hahn & David Root (Addgene plasmid # 23496). pPET‐PKR/PPase was a gift from James Cole (Addgene plasmid # 42934). pRK793 was a gift from David Waugh (Addgene plasmid # 8827). pmTurquoise2‐LAMP1 was a gift from Pablo Rivera‐Fuentes (Addgene plasmid # 110948). pEGFP plasmid (pc3DNA) was purchased from Addgene. Emerin pEGFP‐N2 (588) was a gift from Eric Schirmer (Addgene plasmid # 61985). FITC and Cy3‐labelled plasmid DNA was obtained from Mirus Bio LLC (Madison, WI).

### Cells

Chemically competent *E. coli* DH5alpha and BL21(DE3)pLysS cells were purchased from Promega AG (Dübendorf, Switzerland). HeLa (ATCC CCL‐2) cells were obtained from ATCC (Manassas, VA).

### Chemicals and Consumables

QIAprep Spin Miniprep Kit was obtained from Qiagen (Hilden, Germany). FastDigest DNA restriction enzymes and synthesized double stranded DNA sequences (GeneArt gene synthesis service) were purchased from Thermo Fisher Scientific (Waltham, MA). DNA oligonucleotides were synthesized by Microsynth AG (Balgach, Switzerland). Isopropyl *β*‐D‐1‐thiogalactopyranoside (IPTG), lysozyme, polysorbate 20, EDTA, MES were obtained from AppliChem (Darmstadt, Germany). Na_2_HPO_4_, KH_2_PO_4_, imidazole, Tris‐base, Hoechst 33342, heparin‐agarose, Triton X‐100, KCl, Mowiol 4‐88, paraformaldehyde, Coomassie Brilliant Blue G‐250, ethanol, DTT, hydrogen peroxide, heparan sulfate, polyethyleneimine, bafilomycin A1, sodium dodecyl sulfate, acrylamide, and bovine serum albumin were obtained from Sigma Aldrich Chemie GmbH (Buchs, Switzerland). NaCl, glycine, and glycerol were obtained from Fisher Scientific (Reinach, Switzerland). MgCl_2_ and ammonium molybdate were obtained from ABCR GmbH (Karlsruhe, Germany). UltraPure agarose, FastDigest Green Buffer (10×), FBS, penicillin‐streptomycin (10 000 U mL^–1^), Trypsin, LP3000, Life Cell Imaging Solution, DiI, DMEM high glucose GlutaMAX Supplement and PBS (pH 7.4) were obtained from Thermo Fisher Scientific (Waltham, MA). Acetic acid was obtained from VRW International GmbH (Dietikon, Switzerland). GelRed DNA dye was ordered from Biotium (Hayward, CA). Malachite green was obtained from Bender and Hobein AG (Zürich, Switzerland). POPC was ordered from AdipoGen Life Sciences (San Diego, CA). Calf intestinal phosphatase, T4 DNA ligase buffer were obtained from New England Biolabs (Ipswitch, MA). Protease inhibitor cocktail was obtained from Sigma Aldrich Chemie GmbH (Buchs, Switzerland) and Ni‐NTA agarose from Qiagen (Germantown, MD).

### Cloning

All constructs were generated with FastDigest restriction enzyme‐based cloning into the backbone pET His6 TEV LIC expression vector (1B). The plasmid constructs were amplified in *E. coli* DH5*α*, purified by QIAprep Spin Miniprep Kit (Qiagen, Hilden, Germany), and sequenced over the complete coding sequence (Microsynth AG, Balgach, Switzerland). Correct constructs were then transformed into chemically competent BL21(DE3)pLysS cells.

The His_6_‐TFAM‐VRK1 lambda phosphatase plasmid was generated with 3 inserts that were amplified by PCR; human TFAM (uniport: TFAM_HUMAN), human VRK1 (uniport: VRK1_HUMAN), and *Escherichia phage* lambda phosphatase (uniport: PP_LAMBD). The TFAM insert was produced using the primers 5′‐CGCGGATCCATGTCATCTGTCTTGGCAA‐3′ and 5′‐ATAGTTTAGCGGCCGCTTGATCCACCGGAACACTCCTCAGCACCATA‐3′ from the template plasmid Human_TFAM_NoMTS_pET28 and digested with BamHI and NotI. The VRK1 insert was amplified with the primers 5′‐TATAGAATGCGGCCGCTCCGGTATGCCTCGTGTAAA‐3′ and 5′‐CGGGGTACCTTACTTCTGGACTCTCTTTCTGG‐3′ from the template plasmid pDONR223‐VRK1 and digested with NotI and KpnI. Finally, the RBS‐lambda phosphatase fragment was generated with the primers 5′‐CGGGGTACCACACATGTTAGAGCCCTTC3′ and 5′‐CCGCTCGAGTTCCTTTCGGGCTTTGTTA‐3′ from the template pPET‐PKR/PPase and digested with KpnI and XhoI. The three fragments were ligated into the cloning vector which was previously linearized with BamHI and XhoI.

His_6_‐cysTFAM‐VRK1 lambda phosphatase: The TFAM gene in the original construct above was replaced with the TFAM mutant A105C, V109C. The insert was generated by GeneArt gene synthesis (Table S2, Supporting Information) and inserted at the restriction sites XbaI and NotI. Analogously, the constructs His_6_‐TFAM(A105C)‐VRK1, His_6_‐TFAM(V109C)‐VRK1 and His_6_‐TFAM(A105S,V109S)‐VRK1 were cloned by replacing wild‐type TFAM in the original construct with the mutant sequences (Table S2, Supporting Information).

The kinase dead constructs TFAM‐deadVRK1 and cysTFAM‐deadVRK1 were generated by replacing the VRK1 sequence in the TFAM‐VRK1 or cysTFAM‐VRK1 lambda phosphatase construct, respectively. The original plasmid was digested with NotI and XhoI. The deadVRK1 insert was generated by fusion PCR using the primers 5′‐TATAGAATGCGGCCGCTCCGGTATGCCTCGTGTAAA‐3 and 5′‐ATGGAGCTATCAAGGCCTCAAATCTTCTTCTG‐3 for fragment A and 5′‐TTTGAGGCCTTGATAGCTCCATGCACATACTCAT‐3 and 5′‐CCGCTCGAGTTACTTCTGGACTCTCTTTCT‐3 for fragment B with the original VRK1 sequence as a template. The gene fragments containing the point mutation were then fused together by PCR with the primers 5′‐TATAGAATGCGGCCGCTCCGGTATGCCTCGTGTAAA‐3′ and 5′‐CCGCTCGAGTTACAACTTCTGGACTCTCTTTCT‐3′. The fused insert was digested with NotI and XhoI and ligated into the linearized vector His6‐TFAM‐VRK1 or His6‐cysTFAM‐VRK1 lambda phosphatase.

TFAM, cysTFAM, and cysTFAM‐NLS: The wild‐type TFAM sequence was amplified by PCR from the template Human_TFAM_NoMTS_pET28 with the primers 5′‐ACCCAAGCTTCGATGTCATCTGTCTTGGCAA‐3′ and 5′‐CCGCTCGAGTTAACACTCCTCAGCACCATA‐3′. The insert was ligated into the expression plasmid at the restriction sites HindIII and XhoI. The cysTFAM gene (Tables S2.3, Supporting Information) was generated by GeneArt synthesis and inserted into the expression plasmid at the restriction sites HindIII and XhoI. CysTFAM‐NLS was cloned as TFAM, but with the reverse primer 5′‐CCACTCGAGTTACACCTTCCTCTTCTTCTTGGGACCCGAACACTCCTCAGCACCATA‐3′ and the cysTFAM construct as PCR template.
PLC‐TFAM constructs: The MBP insert was cloned using the primers 5′‐GATAACAATTCCCCTCTAG‐3′ and 5′‐GAGCTGCAGCCCGAGGTTGTTGTTATTG‐3′ from the template plasmid pRK793 and digested with XbaI and PstI. The PLC (uniport: PHLC_LISMO) gene was synthesized by GeneArt gene synthesis with terminal PstI and BamHI restriction sites (Table S2.4, Supporting Information). The TFAM gene was amplified from the plasmid Human_TFAM_NoMTS_pET28 using the primers 5′‐CTACTGCAGGCTTCAAGCGAAAATCTGT‐3′ and 5′‐CCGCTCGAGTTAACACTCCTCAGCACCATA‐3′. Subsequently, the three fragments were ligated into the expression plasmid that was previously linearized with the restriction enzymes XbaI and XhoI.

The PLC variant constructs MBP‐PLC‐cysTFAM containing a phospholipase‐dead PLC mutant (W52G) was obtained by PCR with the primers 5′‐CATCTGCAGGAAAATCTGTATTTCCAAGGGAGCGCGGACAATCCG‐3′ and 5′‐CCGCTCGAGTTAACACTCCTCAGCACCATA‐3′ from the original construct. The sequence was inserted in the MBP‐PLC‐TFAM plasmid at the restriction sites PstI and XhoI. MBP‐PLC‐cysTFAM was cloned by replacing the wild‐type TFAM sequence in the original plasmid with the double cysteine mutant (TFAM A105C, V109C). The cysTFAM sequence was synthesized by GeneArt gene synthesis as above and inserted at the restriction sites BamHI and XhoI.

### Protein Expression

All the TFAM proteins were expressed and purified according to the same method,^[^ ^40^ ^]^ with some modifications. The proteins were expressed in *E. coli* (DE3)pLysS cells. First the cells were grown to an OD_600_ of 0.5 at 37 °C with shaking at 250 rpm (Universal Shaker SM‐30, Huber & Co AG, Reinach, Switzerland). The bacteria were induced with IPTG (0.4 × 10^–3^ m) at 30 °C, 250 rpm for 4 h. Subsequently, the bacteria were pelleted at 4000 × *g*, 4 °C for 10 min (Heraeus Megafuge 16R centrifuge, Thermo Scientific, Waltham, MA). The supernatant was discarded, the pellet was frozen in liquid nitrogen and stored at −20 °C. The next day, the pellet was thawed on ice and resuspended with lysis buffer (sodium hydrogen phosphate (Na_2_HPO_4,_ 5 × 10^–3^ m), potassium dihydrogen phosphate (KH_2_PO_4,_ 1 × 10^–3^ m), NaCl (70 mm), potassium chloride (KCl, 500 × 10^–3^ m), glycerol (10%), lysozyme (1 mg mL^–1^), protease inhibitor cocktail, DTT (1 × 10^–3^ m), pH 7.4). The suspension was incubated for 30 min at room temperature. Then, the sample was cooled on ice and sonicated (FB705 sonicator, Fisher Scientific) with a total pulse time of 3 min, 5 s pulses, 10 s breaks on ice. Genomic DNA was precipitated by adding branched polyethyleneimine (0.1% (w/v)) with a molecular weight of 25 kDa. The cellular debris was removed by centrifugation at 20 000 × *g* for 45 min, 4 °C. The supernatant was collected, filtered through a 0.2‐µm filter, and loaded on a Ni‐NTA agarose resin, that was pre‐equilibrated with column loading buffer (lysis buffer supplemented with imidazole (10 × 10^–3^ m), but without lysozyme and protease inhibitors). The column was thoroughly washed with at least 10 resin volumes of loading buffer, followed by 5 resin volumes of loading buffer supplemented with imidazole (20 × 10^–3^ m). Eventually, the protein was eluted with loading buffer containing imidazole (250 × 10^–3^ m). The protein concentration was estimated by spectrophotometry at 280 nm (NanoPhotometer Pearl, Implen GmbH, Munich, Germany). The extinction coefficients and molecular masses of all the constructs can be found in Table S3, Supporting Information. The physicochemical parameters of all proteins were calculated using the ProtParam (ExPASy, Bioinformatics Resource Portal, Switzerland) online tool. The PLC proteins containing a cleavable MBP solubility tag were incubated for at least 4 h with a 1:1 (w/w) ratio of TEV protease (purified from the plasmid pRK793 according to ref. [41]) in column elution buffer.

For the second purification step, a heparin‐agarose column with 1 mL resin volume was prepared and equilibrated with heparin buffer (PBS, DTT (1 × 10^–3^ m)). The protein samples were diluted fourfold with ddH_2_O to decrease the salt concentration below 300 × 10^–3^ m, and immediately loaded on the heparin column. The column was then washed with 10 column volumes of heparin buffer. Eventually, the protein was eluted with heparin elution buffer (Na_2_HPO_4_ (5 mm), KH_2_PO_4_ (1 × 10^–3^ m), NaCl (1 m), glycerol (20%), DTT (1 × 10^–3^ m), magnesium chloride (MgCl_2,_ 5 × 10^–3^ m)). A buffer exchange was performed by ultrafiltration (Amicon Ultra‐15, PLGC Ultracel‐PL Membrane, 10 kDa, Sigma Aldrich, St Louis, MO) at 4 °C, 4000 × *g* into heparin elution buffer with NaCl (70 × 10^–3^ m). The protein concentrations were again estimated by spectrophotometry. Protein purity was analyzed by sodium dodecyl sulfate (SDS)‐PAGE with Coomassie Brilliant Blue G‐250 staining and subsequent densiometric analysis with the software ImageJ. SDS‐PAGEs of the main proteins of the study are shown in Figure S14, Supporting Information.

### Detection of TFAM DNA‐Binding Activity

An agarose gel (0.8%) was prepared with 1× TAE buffer (Tris‐base (40 × 10^–3^ m), acetate (20 × 10^–3^ m), EDTA (1 × 10^–3^ m), supplemented with GelRed DNA dye (1:20 000)). The samples were prepared in 1.5 mL test tubes in a total volume of 10 µL. First, PBS buffer was added, followed by the proteins of interest and finally pEGFP plasmid DNA (100 ng). The tubes were mixed by tapping and incubated at room temperature for 30 min. Subsequently, FastDigest Green buffer (10×, 2 µL) was added to each tube and the samples (11 µL each) were loaded on the gel. Electrophoresis was performed in 1× TAE buffer at 100 mA for 45–60 min in an electrophoresis chamber (Sub‐Cell GT Cell, Bio‐Rad, Hercules, CA). The DNA was visualized on a ChemiDoc MP Gel reader (Bio‐Rad).

### Protection of DNA from BAF‐Dependent Clustering by VRK1

The assay was performed according to the gel mobility shift protocol above with the differences that T4 ligase buffer (containing ATP) was used instead of PBS and BAF (1 × 10^–6^ m, purified according to ref. [20]) was added to the indicated samples after 20 min of TFAM/DNA co‐incubation. The samples were incubated for another 5 min at room temperature and then loaded on an agarose gel (0.8%) with FastDigest Green loading buffer.

### PLC Activity Test—Malachite Green Assay

The assay was modified from ref. [28]. The malachite green solution was prepared by mixing ammonium molybdate (4.2%) in HCl (4 m) with malachite green solution (0.045%) in a ratio 1:4 (v/v). The mixture was filtered through a 0.2‐µm filter and supplemented with polysorbate 20 (1%) just before use. A stock solution of POPC was prepared by dissolving POPC (12 × 10^–3^ m) in ethanol (12 µL). Triton X‐100 (23 µL) was added to the lipid solution followed by rapid dilution to a total volume of 1 mL with ddH_2_O to form liposomes.

Subsequently, POPC liposome suspension (50 µL) was mixed with PLC reaction buffer (50 µL, 2‐(N‐morpholino)ethanesulfonic acid (MES, 40 × 10^–3^ m, pH 5.0), NaCl (300 × 10^–3^ m)) and the PLC construct of interest (2 × 10^–6^ m). To test the PLC activity in the presence of pDNA, PLC‐TFAM (1 × 10^–6^ m) was incubated with pDNA (100 ng) for 20 min in PBS in a total volume of 10 µL. From this sample, 5 uL were added to the liposome solution, resulting in a PLC concentration of 0.1 × 10^–6^ m.

After 5 min of incubation at 37 °C, 45 µL of the samples were quenched with Tris buffer (pH 8.0, 15 µL, 2 m) and incubated at 98 °C for 10 min. Then, the samples were centrifuged at 18 000 × *g* for 10 min at 4 °C. The supernatant (50 µL) was transferred to a 96‐well plate (Microplate, 96‐well, polystyrene, flat, transparent, Greiner bio ‐one, Kremsmünster, Austria), followed by the addition of calf intestinal phosphatase (0.5 µL) to each well. The plate was incubated for 30 min at 37 °C. The samples (10 µL) were mixed with ddH_2_O (65 µL) and malachite green‐polysorbate 20 solution (75 µL). The samples were again incubated for 30 min at room temperature. Eventually, the absorbance was measured on a TECAN infinite M200 plate reader (Tecan AG, Mannedorf, Switzerland) at 620 nm. A standard curve was generated with NaHPO_4_ (1–30 × 10^–6^ m) which was used to determine the amount of released phosphate by the enzyme and to calculate its activity in × 10^–6^ m released phosphate per min and per × 10^–6^ m PLC construct. The background activity (buffer control) was subtracted from each value.

### TEM

PEGFP DNA (10 ng µL^–1^) was mixed with the indicated concentrations of the TFAM protein constructs in a total volume of 10 µL PBS. After 30 min of incubation at 25 °C, the samples were diluted to 40 µL with PBS and stored on ice. Carbon coated grids (Quantifoil, Großloebichau, GE) were positively glow discharged (Pelco easiGlow, Ted Pella Inc., Redding, CA) and placed inversely on 10 µL sample drops on parafilm and the particles were allowed to adsorb for 30 min. Subsequently, excess fluid was removed with filter paper and the grids were washed three times in double distilled water for ≈1 s by slight lateral movements on the surface of the water. The wet samples were stained in uranyl acetate (2%) for 1 s followed by a second step for 15 s. Excess moisture was drained with filter paper and the imaging of the air‐dried grids was performed in a TEM Morgagni 268 (Thermo Fisher Scientific) operated at 100 kV.

### NTA

The DNA/protein particles were formed in 2 mL test tubes by adding PBS buffer with the proteins of interest, followed by pEGFP DNA (100 ng) in a 10 µL volume. After 30 min of incubation at room temperature, the sample was diluted 1:200 (v/v) to a final concentration of 10^7^ particles mL^–1^ with PBS. Size profile and zeta potential measurements were performed at 11 positions with a sensitivity of 85, shutter of 100, and frame rate of 30 on a ZetaView instrument equipped with a CMOS camera and a 405 nm laser (Particle Metrix GmbH, Meerbusch, Germany). Size profile data was smoothed over 4 neighbors with a second order smoothing polynomial function using GraphPad Prism software (version 8.0, GraphPad Software Inc., San Diego, CA).

### Cell Culture

HeLa cells were cultured in full growth medium (DMEM, supplemented with FBS (10%) and penicillin‐streptomycin (1%)) at typical mammalian cell culture conditions (37 °C, 5% CO_2_, humidified atmosphere). The cells were used for experiments in passage number 2–30 and tested negative for mycoplasma contamination (MycoAlert Kit, Lonza AG, Basel, Switzerland). In preparation for a transfection experiment the cells were trypsinized and seeded to reach 90–100% confluency on the day of transfection.

### Particle Formation and Transfection

Particle formation was performed in 1.5 mL test tubes in a total volume of 10–100 µL. First, PBS (or FBS to a final concentration of 80%) was added followed by the proteins of interest in the indicated concentrations and, eventually, the plasmid DNA (10 ng µL^–1^). The samples were incubated for 30 min. Subsequently, 10 µL sample was added to one well of a 24‐well tissue culture plate (TPP Techno Plastic Products AG, Trasadingen, Switzerland) resulting in a final DNA concentration of 200 ng mL^–1^ growth medium. Note that a medium exchange was performed 30 min prior to transfection in which the cell medium was renewed to perform transfections in 10% FBS. Alternatively, the cell medium was replaced with serum which resulted in approximate FBS concentrations of 95% or 99% during transfection, depending on whether the added TFAMoplexes were formed in PBS or serum, respectively. The cells were incubated with the particles for 30 min (or as indicated) under standard culture conditions. Subsequently, the wells were washed three times with PBS (or as indicated) and were further incubated in full growth medium. LP3000 was used as a positive control according to the manufacturer protocol. In brief, LP3000 (3 µL) reagent was diluted in OptiMEM I (50 µL, Thermo Fisher, Waltham, MA) in test tube A. In test tube B, P3000 reagent (2 µL), and plasmid DNA (1 µg) were added to OptiMEM I (50 µL). The content of tube B was then mixed with tube A and incubated for 10 min at room temperature. Subsequently, 10 µL lipoplexes were applied per well of a 24‐well plate containing 0.5 mL cell medium (or FBS). To form LP3000 lipoplex in serum, 80% FBS (with 20% OptiMEM) was used for particle formation instead of pure OptiMEM. XtremeGene‐9 transfection was conducted according to the manufacturer protocol with a 3:1 ratio of transfection agent to DNA, but particle formation was performed in 80% FBS (with 20% OptiMEM) instead of OptiMEM.

### Bafilomycin A1 Treatment

The cells were preincubated with 500 × 10^–9^ m bafilomycin A1 in complete growth medium and normal growth conditions. After transfection the cells were washed three times with PBS and further incubated with 500 × 10^–9^ m bafilomycin A1 in complete growth medium for additional 6 h. Eventually, the cells were washed and incubated in growth medium without inhibitor until the time point of analysis.

### Inverted Setup Transfections^[^ ^42^ ^]^

The cells were seeded on plastic cover slips to reach a confluency of 90–100% on the day of transfection. The DNA/protein particles were applied to FBS (0.5 mL) on a separate 24‐well plate and mixed. Then the coverslip was placed on top of the medium with the cells facing toward the bottom of the dish. After 30 or 120 min of incubation at 37 °C, 5% CO_2_ the coverslips were put on the bottom of a fresh 24‐well plate, washed three times with PBS, and further incubated in full growth medium until the time point of analysis (22–24 h after transfection).

### Fluorescence‐Activated Cell Sorting (FACS)

HeLa cells growing on a 24‐well plate were washed four times with PBS, and subsequently trypsinized for 2 min at 37 °C. The cells were collected in 4 °C complete growth medium and pelleted by centrifugation at 300 × *g* for 10 min at 4 °C. The pellet was resuspended in ice cold FACS buffer (150 µL, PBS, EDTA (2 × 10^–3^ m), bovine serum albumin (0.05%)) and transferred to a Costar round‐bottom 96‐well assay plate (Corning, New York, NY) for FACS analysis (CytoFLEX Flow Cytometer, Beckman Coulter Life Sciences, Nyon, Switzerland). For each measurement, 10 000 cells were collected and analyzed according to their fluorescein isothiocyanate (FITC) fluorescence (excitation at 488 nm) using the FlowJo software (Tree Star Inc., Ashland, OR).

### Cell Viability Assay

HeLa cells were seeded on a 96‐well tissue culture plate (TPP Techno Plastic Products AG) with 5000 cells per well. The next day, DNA/TFAM particles were formed as described above and added to the cells in the indicated concentrations (expressed in ng DNA per mL complete culture medium, 10% FBS). After 17 h of incubation, CellTiter 96 Aqueous One Solution Reagent (20 µL, Promega) was given to each well. As a positive control hydrogen peroxide (10 × 10^–3^ m) was used. The plate was incubated for 1.5 h at 37 °C, 5% CO_2_, humified atmosphere. Subsequently, absorbance was recorded at 490 nm on a TECAN infinite M200 plate reader. The data were normalized with respect to the “DNA only” negative control.

### Confocal Laser Microscopy

The cells were seeded on glass cover slips to reach 50% confluency on the day of transfection. The particles were formed as described above with FITC‐labelled plasmid DNA and applied to the cells in DMEM supplemented with FBS (10%) for the indicated time periods under standard incubation conditions. Afterward, the coverslips were washed four times with 37 °C PBS and, as indicated, with heparan sulfate (40 mg mL^–1^) or a pH 3 glycine buffer. Then the cells were fixed with paraformaldehyde solution (4%) in PBS for 10 min at room temperature. The paraformaldehyde was discarded and the coverslips were washed three times with PBS. Subsequently, the cells were incubated simultaneously with Hoechst 33342 (1 µg mL^–1^) and 1,1”‐dioctadecyl‐3,3,3”,3′‐tetramethylindocarbocyanine perchlorate (DiI) (5 µg mL^–1^) in PBS for 10 min at room temperature. The coverslips were again washed three times with PBS and eventually mounted on glass specimen with a drop of Mowiol 4–88.

Confocal laser microscopy was performed on a Visitron Spinning Disk (Puchheim, Germany) using an APO Plan 100× objective (MA848 Semi APO Plan objective F100X oil, Meiji Techno, San Jose, CA). Sample excitation was performed at 405, 488, and 561 nm with illumination intensities of 53, 80, and 118, respectively. Z‐stacks were acquired with a slice‐thickness of 0.3 µm. The images were processed and analyzed with the software ImageJ.

### Non‐Reducing SDS‐PAGE Analysis of TFAM and cysTFAM Proteins

The proteins (1 × 10^–6^ m) were mixed with 100 ng pDNA or without pDNA in PBS and incubated for 30 min at room temperature. Then the proteins were denaturated with gel loading buffer (200 × 10^–3^ m Tris‐HCl, pH 6.8, 2% SDS, 10% glycerol, 10 × 10^–3^ m EDTA) and loaded on a 12% non‐reducing SDS‐PAGE (1 µg protein per lane). The gel was subsequently stained with coomassie blue and imaged.

### Statistics

Statistical analysis was performed using the GraphPad Prism software version 8.0. All data are generally represented as mean with SD of at least 3 independent experiments. Significance was tested by one‐way ANOVA combined with the Tukey's multiple comparisons methods.

## Conflict of Interest

The authors declare no conflict of interest.

## Author Contributions

M.B. elaborated the transfection concept. He designed and produced the protein constructs and performed the experiments under the supervision of J.‐C.L. S.K. produced various protein batches and set up the malachite green assay for PLC activity measurements under the supervision of J.‐C.L. and M.B. The article was written, and the figures prepared by M.B. with data analysis, text editing, and proof‐reading by J.‐C.L. All authors reviewed the manuscript.

## Supporting information

Supporting Information

Supporting Information

Supporting Information

Supporting Information

Supporting Information

Supporting Information

Supporting Information

## Data Availability

The data that supports the findings of this study are available in the supplementary material of this article.

## References

[1] X. M. Anguela , K. A. High , Annu. Rev. Med. 2019, 70, 273. 30477394 10.1146/annurev-med-012017-043332

[2] B. Shi , M. Zheng , W. Tao , R. Chung , D. Jin , D. Ghaffari , O. C. Farokhzad , Biomacromolecules 2017, 18, 2231. 28661127 10.1021/acs.biomac.7b00803

[3] D. B. Thompson , J. J. Cronican , D. R. Liu , Methods Enzymol. 2012, 503, 293. 22230574 10.1016/B978-0-12-396962-0.00012-4 PMC3505079

[4] Z. Kang , Q. Meng , K. Liu , J. Mater. Chem. B 2019, 7, 1824. 32255045 10.1039/c8tb03124j

[5] C. T. Campbell , J. E. Kolesar , B. A. Kaufman , Biochim. Biophys. Acta 2012, 1819, 921. 22465614 10.1016/j.bbagrm.2012.03.002

[6] U. Muthurajan , F. Mattiroli , S. Bergeron , K. Zhou , Y. Gu , S. Chakravarthy , P. Dyer , T. Irving , K. Luger , Methods Enzymol. 2016, 573, 3. 27372747 10.1016/bs.mie.2016.01.002 PMC5098222

[7] B. A. Kaufman , N. Durisic , J. M. Mativetsky , S. Costantino , M. A. Hancock , P. Grutter , E. A. Shoubridge , Mol. Biol. Cell 2007, 18, 3225. 17581862 10.1091/mbc.E07-05-0404 PMC1951767

[8] H. B. Ngo , G. A. Lovely , R. Phillips , D. C. Chan , Nat. Commun. 2014, 5, 3077. 24435062 10.1038/ncomms4077 PMC3936014

[9] C. Kukat , K. M. Davies , C. A. Wurm , H. Spahr , N. A. Bonekamp , I. Kuhl , F. Joos , P. L. Polosa , C. B. Park , V. Posse , M. Falkenberg , S. Jakobs , W. Kuhlbrandt , N. G. Larsson , Proc. Natl. Acad. Sci. USA 2015, 112, 11288. 26305956 10.1073/pnas.1512131112 PMC4568684

[10] K. Kim , J. S. Han , H. A. Kim , M. Lee , Biotechnol. Lett. 2008, 30, 1331. 18347754 10.1007/s10529-008-9695-4

[11] B. Kim , J. H. Song , M. Lee , J. Drug Targeting 2014, 22, 739. 10.3109/1061186X.2014.916711 24830301

[12] Y. Shen , H. Peng , J. Deng , Y. Wen , X. Luo , S. Pan , C. Wu , M. Feng , Int. J. Pharm. 2009, 375, 140. 19442462 10.1016/j.ijpharm.2009.03.040

[13] H. B. Ngo , J. T. Kaiser , D. C. Chan , Nat. Struct. Mol. Biol. 2011, 18, 1290. 22037171 10.1038/nsmb.2159 PMC3210390

[14] A. Grundling , M. D. Gonzalez , D. E. Higgins , J. Bacteriol. 2003, 185, 6295. 14563864 10.1128/JB.185.21.6295-6307.2003 PMC219411

[15] H. Goldfine , N. C. Johnston , C. Knob , J. Bacteriol. 1993, 175, 4298. 8331063 10.1128/jb.175.14.4298-4306.1993 PMC204869

[16] G. van Meer , D. R. Voelker , G. W. Feigenson , Nat. Rev. Mol. Cell Biol. 2008, 9, 112. 18216768 10.1038/nrm2330 PMC2642958

[17] Q. Huang , A. Gershenson , M. F. Roberts , Biochim. Biophys. Acta 2016, 1864, 697. 26976751 10.1016/j.bbapap.2016.03.008 PMC4829451

[18] H. T. Le , G. A. Rao , A. C. Hirko , J. A. Hughes , Mol. Pharmaceutics 2010, 7, 1090. 10.1021/mp900192p 20459116

[19] S. Toita , S. Sawada , K. Akiyoshi , J. Controlled Release 2011, 155, 54. 10.1016/j.jconrel.2010.12.008 21185892

[20] M. Burger , C. Schmitt‐Koopmann , J. C. Leroux , Sci. Rep. 2020, 10, 12301. 32704141 10.1038/s41598-020-69246-x PMC7378220

[21] M. Segura‐Totten , A. K. Kowalski , R. Craigie , K. L. Wilson , J. Cell Biol. 2002, 158, 475. 12163470 10.1083/jcb.200202019 PMC2173821

[22] C. M. Bradley , D. R. Ronning , R. Ghirlando , R. Craigie , F. Dyda , Nat. Struct. Mol. Biol. 2005, 12, 935. 16155580 10.1038/nsmb989

[23] M. Cai , Y. Huang , J. Y. Suh , J. M. Louis , R. Ghirlando , R. Craigie , G. M. Clore , J. Biol. Chem. 2007, 282, 14525. 17355960 10.1074/jbc.M700576200

[24] N. Ibrahim , A. Wicklund , M. S. Wiebe , J. Virol. 2011, 85, 11588. 21880762 10.1128/JVI.00641-11 PMC3209281

[25] N. Ibrahim , A. Wicklund , A. Jamin , M. S. Wiebe , Virology 2013, 444, 363. 23891157 10.1016/j.virol.2013.07.002 PMC3755115

[26] R. Zheng , R. Ghirlando , M. S. Lee , K. Mizuuchi , M. Krause , R. Craigie , Proc. Natl. Acad. Sci. USA 2000, 97, 8997. 10908652 10.1073/pnas.150240197 PMC16810

[27] E. R. Slepkov , A. Pavinski Bitar , H. Marquis , Biochem. J. 2010, 432, 557. 20879990 10.1042/BJ20100557 PMC3469326

[28] P. J. Hergenrother , S. F. Martin , Anal. Biochem. 1997, 251, 45. 9300081 10.1006/abio.1997.2251

[29] Y. Fang , M. Akimoto , K. Mayanagi , A. Hatano , M. Matsumoto , S. Matsuda , T. Yasukawa , D. Kang , Mitochondrion 2020, 53, 99. 32439622 10.1016/j.mito.2020.05.003

[30] G. Farge , M. Mehmedovic , M. Baclayon , S. M. van den Wildenberg , W. H. Roos , C. M. Gustafsson , G. J. Wuite , M. Falkenberg , Cell Rep. 2014, 8, 66. 24981867 10.1016/j.celrep.2014.05.046

[31] M. Sanz‐Garcia , I. Lopez‐Sanchez , P. A. Lazo , Mol. Cell. Proteomics 2008, 7, 2199. 18617507 10.1074/mcp.M700586-MCP200 PMC2577208

[32] L. Andreeva , B. Hiller , D. Kostrewa , C. Lassig , C. C. de Oliveira Mann , D. J. Drexler , A. Maiser , M. Gaidt , H. Leonhardt , V. Hornung , K. P. Hopfner , Nature 2017, 549, 394. 28902841 10.1038/nature23890

[33] S. Caielli , S. Athale , B. Domic , E. Murat , M. Chandra , R. Banchereau , J. Baisch , K. Phelps , S. Clayton , M. Gong , T. Wright , M. Punaro , K. Palucka , C. Guiducci , J. Banchereau , V. Pascual , J. Exp. Med. 2016, 213, 697. 27091841 10.1084/jem.20151876 PMC4854735

[34] J. P. Little , S. Simtchouk , S. M. Schindler , E. B. Villanueva , N. E. Gill , D. G. Walker , K. R. Wolthers , A. Klegeris , Mol. Cell. Neurosci. 2014, 60, 88. 24769106 10.1016/j.mcn.2014.04.003

[35] M. W. Julian , G. Shao , Z. C. Vangundy , T. L. Papenfuss , E. D. Crouser , PLoS One 2013, 8, e72354. 23951313 10.1371/journal.pone.0072354 PMC3741150

[36] S. Kobayashi , T. Koujin , T. Kojidani , H. Osakada , C. Mori , Y. Hiraoka , T. Haraguchi , Proc. Natl. Acad. Sci. USA 2015, 112, 7027. 25991860 10.1073/pnas.1501235112 PMC4460496

[37] X. Wang , N. Le , A. Denoth‐Lippuner , Y. Barral , R. Kroschewski , Proc. Natl. Acad. Sci. USA 2016, 113, 7177. 27298340 10.1073/pnas.1606091113 PMC4932973

[38] P. Heller , D. Hobernik , U. Lachelt , M. Schinnerer , B. Weber , M. Schmidt , E. Wagner , M. Bros , M. Barz , J. Controlled Release 2017, 258, 146. 10.1016/j.jconrel.2017.05.012 28501672

[39] S. Bauhuber , C. Hozsa , M. Breunig , A. Gopferich , Adv. Mater. 2009, 21, 3286. 20882498 10.1002/adma.200802453

[40] A. Ramachandran , U. Basu , S. Sultana , D. Nandakumar , S. S. Patel , Nucleic Acids Res. 2017, 45, 861. 27903899 10.1093/nar/gkw1157 PMC5314767

[41] R. B. Kapust , J. Tozser , J. D. Fox , D. E. Anderson , S. Cherry , T. D. Copeland , D. S. Waugh , Protein Eng., Des. Sel. 2001, 14, 993. 10.1093/protein/14.12.993 11809930

[42] S. Bisso , S. Mura , B. Castagner , P. Couvreur , J. C. Leroux , Eur. J. Pharm. Biopharm. 2019, 142, 142. 31220571 10.1016/j.ejpb.2019.06.013

