# Supplementary material for: The TFAMoplex—Conversion of the Mitochondrial Transcription Factor A into a DNA Transfection Agent
Source: Adv Sci (Weinh). 2022 Jan 17;9(8):2104987. doi: 10.1002/advs.202104987 (PMC8922101; doi:10.1002/advs.202104987)
Supplement: Supplementary file 1 — Supporting Information [file ADVS-9-2104987-s001.pdf]

ADVANCED SCIENCE
Open Access

Supporting Information

for *Adv. Sci.*, DOI 10.1002/advs.202104987

The TFAMoplex—Conversion of the Mitochondrial Transcription Factor A into a DNA Transfection Agent

*Michael Burger, Seraina Kaelin and Jean-Christophe Leroux**

ADVANCED SCIENCE
Open Access

Supporting Information

for *Adv. Sci.,* DOI: 10.1002/advs.202104987

The TFAMoplex – Conversion of the Mitochondrial Transcription Factor A into a DNA Transfection Agent

*Michael Burger, Seraina Kaelin, Jean-Christophe Leroux**

Supporting Information

The TFAMoplex – Conversion of the Mitochondrial Transcription Factor A into a DNA Transfection Agent

Michael Burger, Seraina Kaelin, Jean-Christophe Leroux*

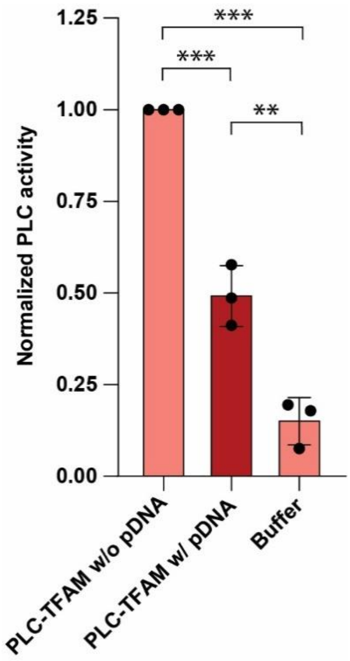

Figure S1. PLC activity measurements by modified malachite green assay. The enzymatic activity of 1 μM PLC-TFAM, both in the presence and absence of 10 ng μL^{-1} plasmid DNA, was measured. Each experiment was normalized with the 'PLC-TFAM w/o DNA' value. Each dot represents the mean of an independent triplicate experiment. Mean ± SD (N=3). **p<0.01, ***p<0.001.

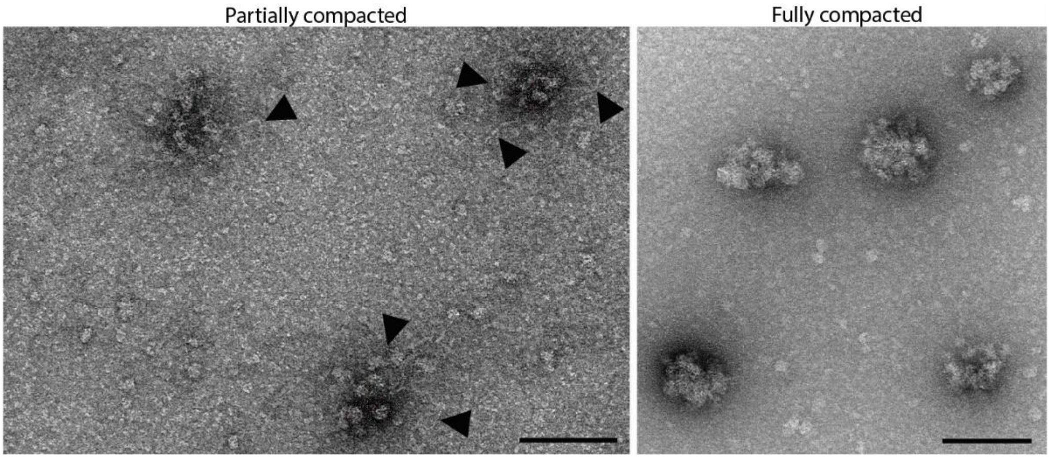

Figure S2. TEM images showing the compaction of pDNA at two PLC-TFAM concentrations, 0.5 μM (left panel) or 1.0 μM (right panel) The black arrow heads indicate free DNA loops. All scale bars 100 nm.

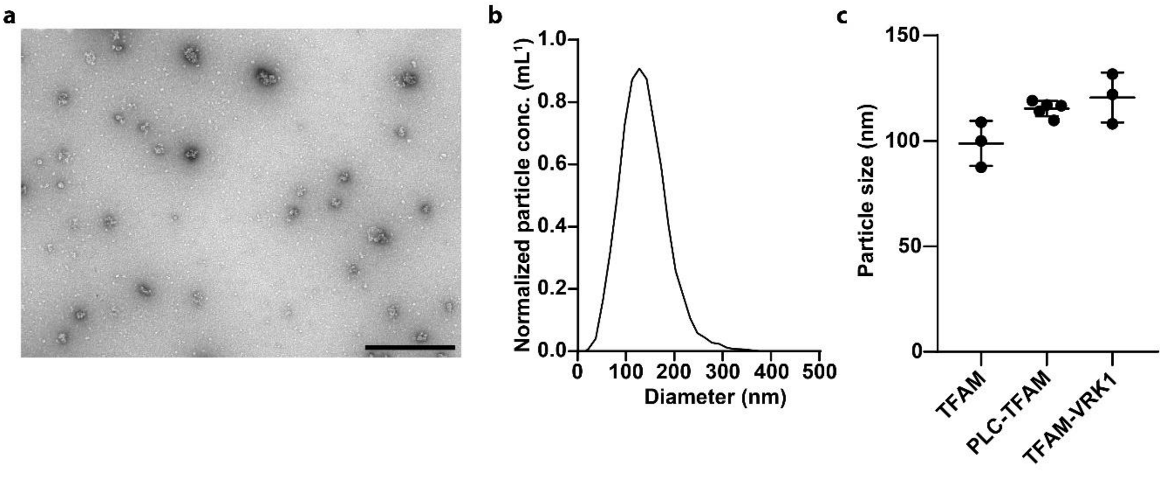

Figure S3. a) TEM image of pDNA complexed with 1 µM TFAM-VRK1. Scale bar: 500 nm. **b)** Representative NTA measurement of DNA/TFAM-VRK1 particles. The data were normalized with respect to the peak value. **c)** DNA/TFAM particle diameter overview. Particle diameters were measured by NTA with the indicated TFAM constructs (1 µM) and pDNA. Mean ± SD (N=3-5).

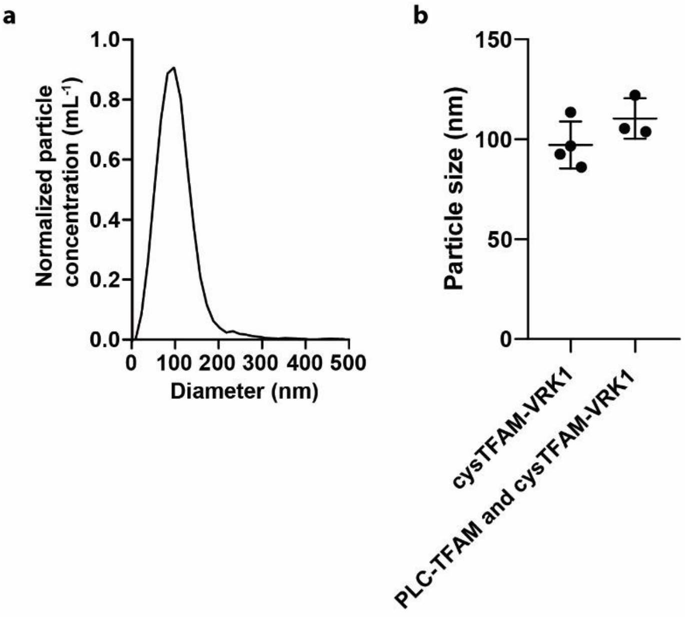

Figure S4. a) Representative NTA measurement of DNA complexed with a 1:1 molar mixture of PLC-TFAM and cysTFAM-VRK1 (1 μM total TFAM). Shown is the particle concentration normalized respective to the peak value. **b)** DNA/TFAM particle diameter overview. Particle diameters were measured by NTA with the indicated TFAM constructs (1 μM) and pDNA. Mean ± SD (N=3-4).

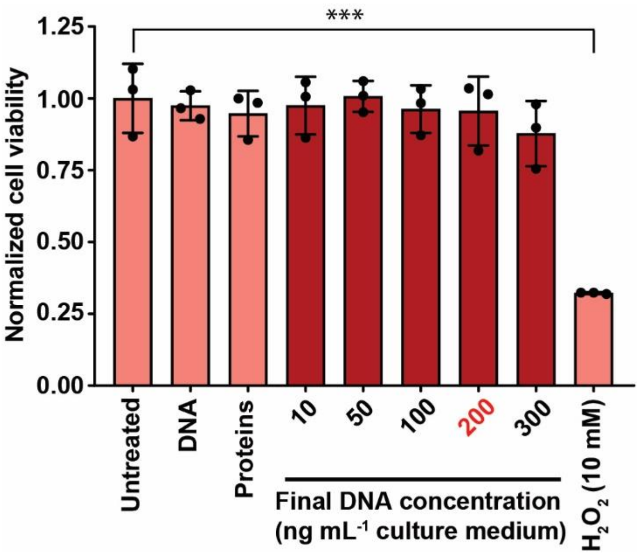

Figure S5. Cell viability assay. HeLa cells were treated for 17 h with the TFAMoplex (cysTFAM-VRK1 and PLC-TFAM) in growth medium supplemented with 10% FBS. The final DNA concentration in the cell growth medium is indicated. The DNA concentration which is used in a standard 30 min transfection experiment is highlighted in red. In the 'protein' control, the protein mixture was applied without DNA in the same concentration as in the 200 ng mL^{-1} DNA condition. Hydrogen peroxide was used as a positive control for cytotoxicity. Data normalized with respect to the untreated cell control. Each dot represents the mean of an independent triplicate experiment. Mean ± SD (N=3), ***$p<0.001$.

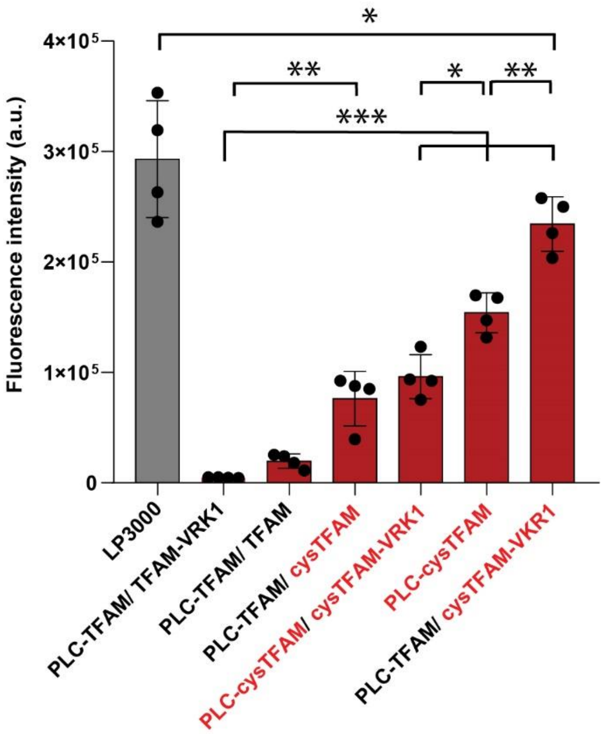

Figure S6. Flow cytometry analysis of HeLa cells transfected with the DNA/TFAM complexes or with LP3000 in 95% FBS. 200 ng pDNA per mL culture medium was used. The DNA/TFAM particles were formed with various TFAM variants in a 1:1 ratio and a total TFAM concentration of 1 μM. Shown are the GFP fluorescence intensities. Each dot represents the mean of an independent triplicate experiment. Mean ± SD (N=4), *$p<0.05$, **$p<0.01$, ***$p<0.001$.

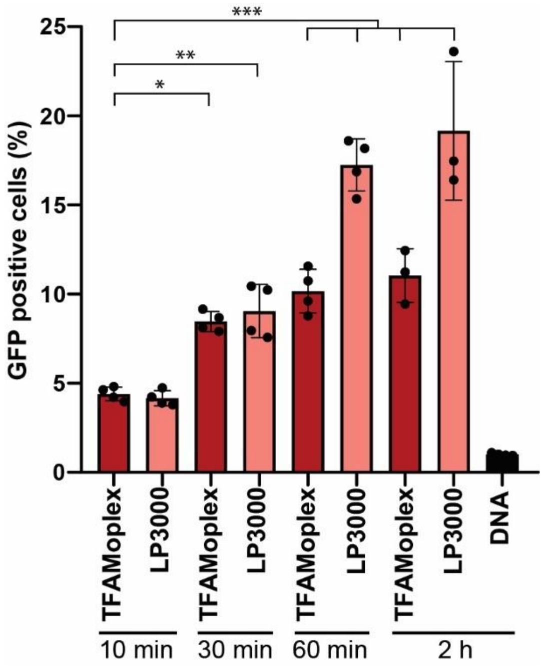

Figure S7. Flow cytometry analysis of HeLa cells 24 h after transfection with TFAMoplex or LP3000 in 95% FBS. 200 ng pDNA per mL culture medium was used for the indicated incubation times. Each dot represents the mean of an independent triplicate experiment. Mean ± SD (N=3-4), *$p<0.05$, **$p<0.01$, ***$p<0.001$.

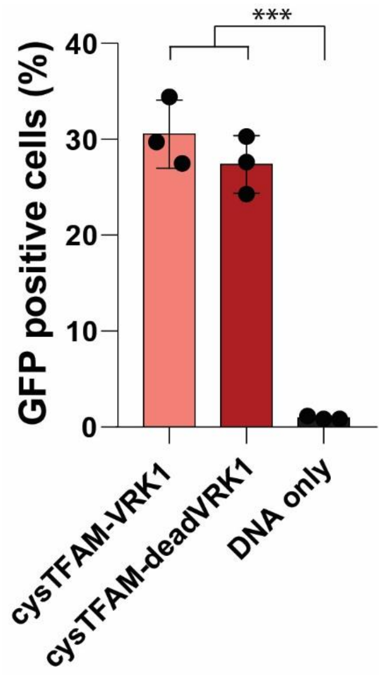

Figure S8. Impact of VRK1 kinase activity on HeLa cell transfection. The pDNA particles were formed in 80% FBS with an equimolar mixture of PLC-TFAM with either cysTFAM-VRK1 or cysTFAM-deadVRK1. The transfection was performed for 30 min in 99% FBS with 200 ng DNA per mL FBS. Analysis performed by FACS. Each dot represents the mean of an independent triplicate experiment. Mean ± SD (N=3). ***$p<0.001$.

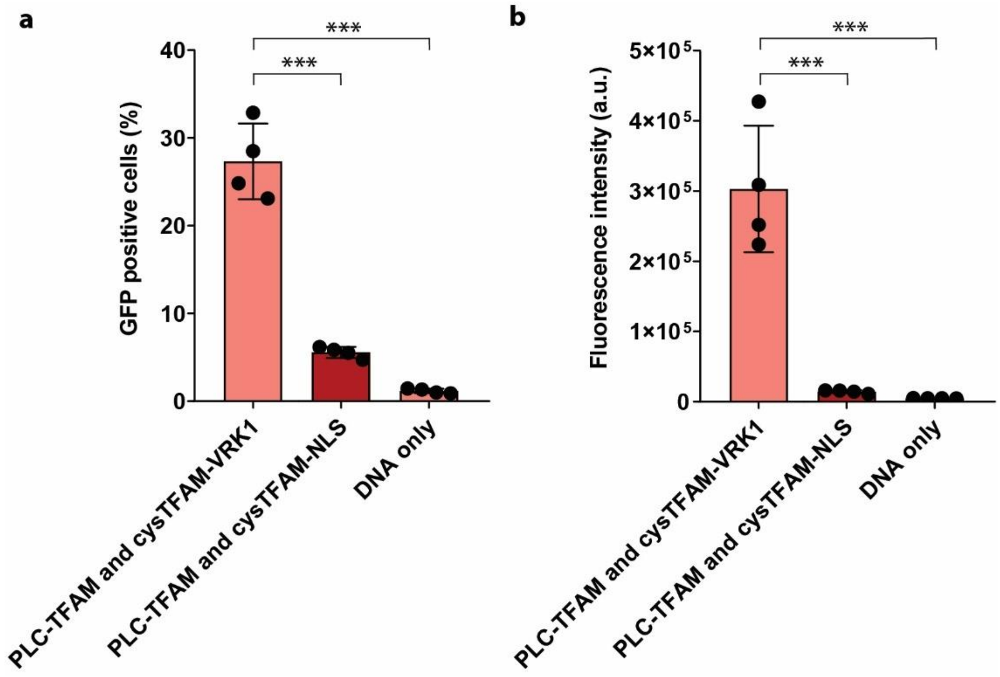

Figure S9. Replacement of VRK1 in cysTFAM-VRK1 with a nuclear localization signal (NLS). The pDNA particles were formed in 80% FBS with an equimolar mixture of PLC-TFAM with either cysTFAM-VRK1 or cysTFAM-NLS. HeLa transfection was performed in 99% FBS for 30 min with 200 ng DNA per mL FBS. Analysis performed 24 h after transfection by FACS. Shown are the percentage of GFP positive cells (a) and the fluorescence intensities (b). Each dot represents the mean of an independent triplicate experiment. Mean ± SD (N=4). ***$p<0.001$.

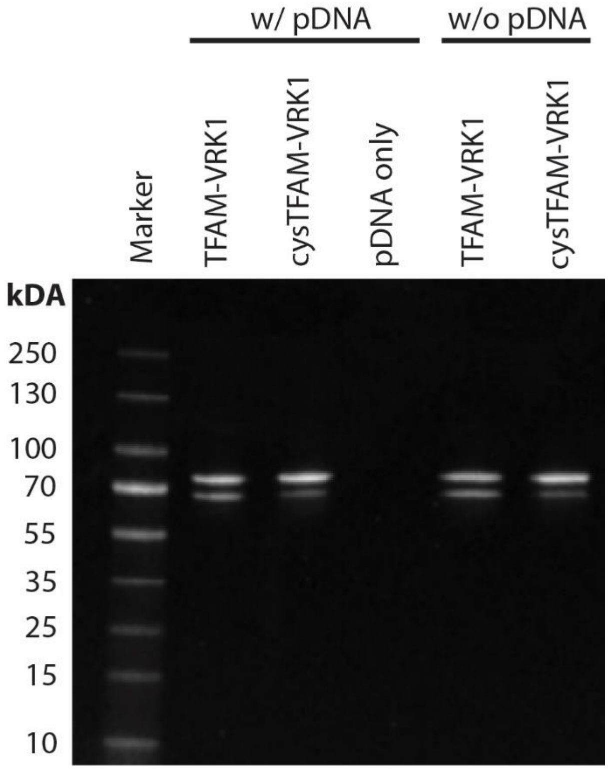

Figure S10. Non-reducing SDS-PAGE analysis of TFAM-VRK1 and cysTFAM-VRK1 proteins. The proteins (1 μM) were mixed with 100 ng pDNA or without pDNA in PBS and incubated for 30 min. Then the proteins were denaturated with gel loading buffer (200 mM Tris-HCl, pH 6.8, 2% SDS, 10% glycerol, 10 mM EDTA) and loaded on a 12% non-reducing SDS-PAGE (1 μg protein per lane). The gel was subsequently stained with Coomassie and imaged. No difference in band mobility was observed, indicating the absence of inter-protein disulfide bridge formation.

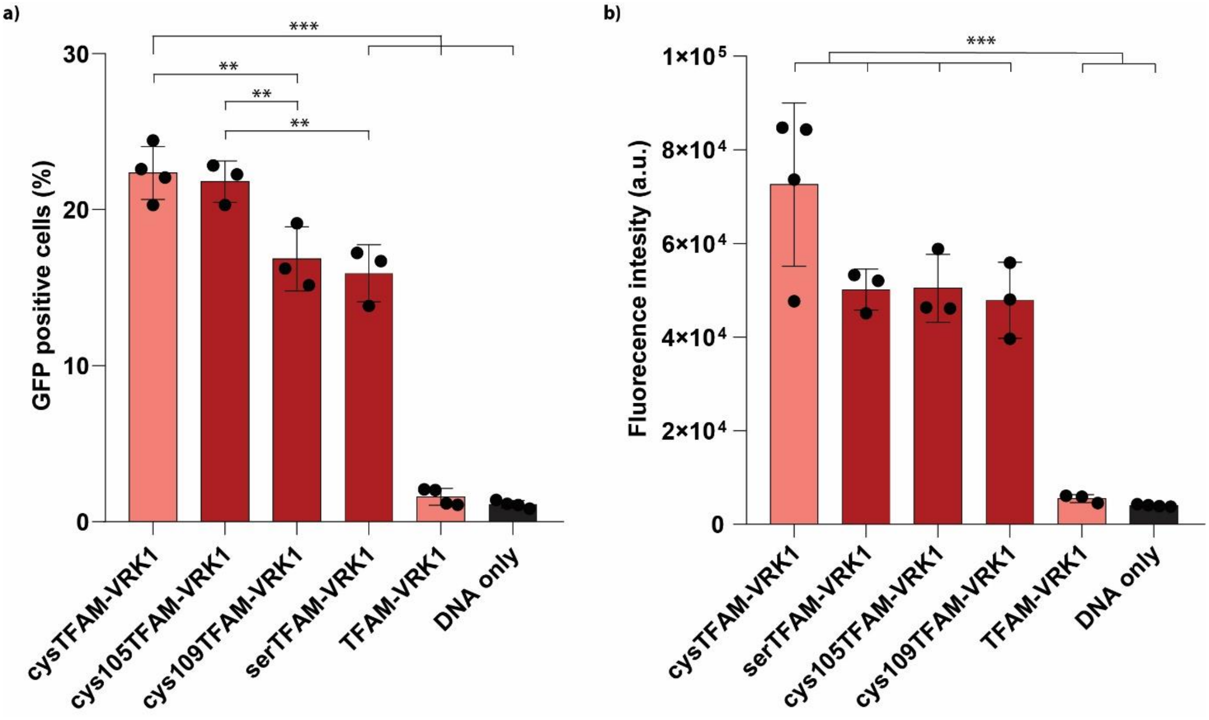

Figure S11. Control constructs serTFAM-VRK1, cys105TFAM-VRK1 and cys109TFAM-VRK1. The pDNA particles were formed with the indicated variant of TFAM-VRK1 in combination with PLC-TFAM and pEGFP in 80% FBS. HeLa transfection was performed in 99% FBS for 30 min with 200 ng DNA per mL FBS. Analysis was performed 24 h after transfection by FACS. Shown are the percentage of GFP positive cells (a) and the mean GFP fluorescence intensities (b). Each dot represents the mean of an independent triplicate experiment. Mean ± SD (N=3-4). **p<0.01, ***p<0.001.

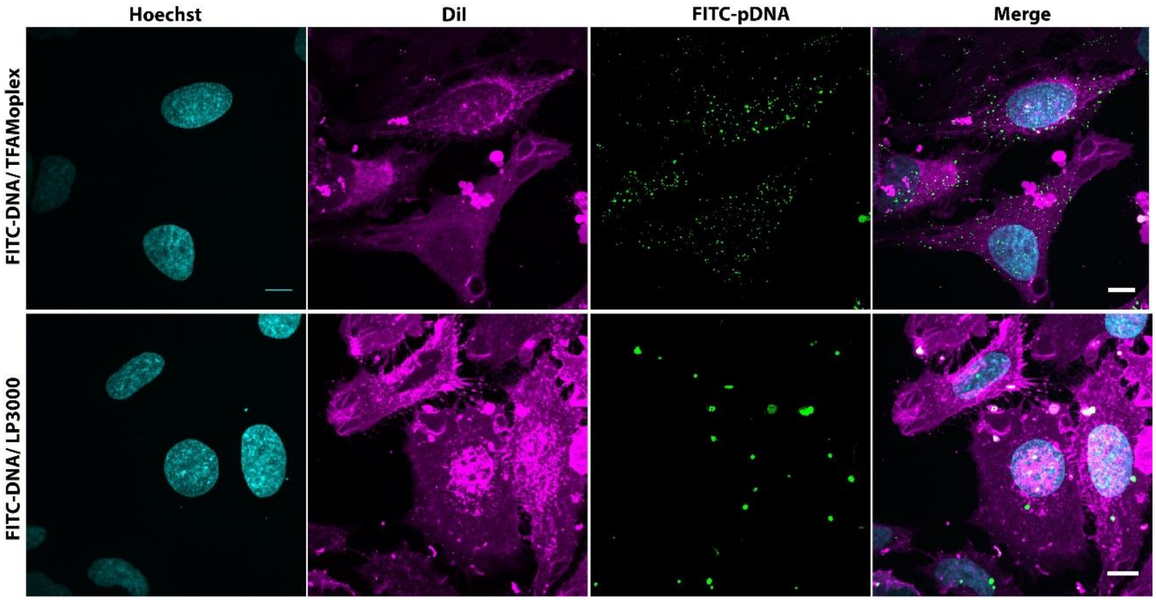

Figure S12. Representative confocal images of HeLa cells 2 h after incubation with FITC-DNA/TFAMoplex or FITC-DNA/LP3000 lipoplexes in growth medium supplemented with 10% serum, normal set-up. Shown is the z-stack projection of maximum signal intensities (a) and a single z-slice. Cyan: Hoechst DNA staining. Magenta: DiI membrane staining. Green: FITC-pDNA. Scale bars: 10 µm.

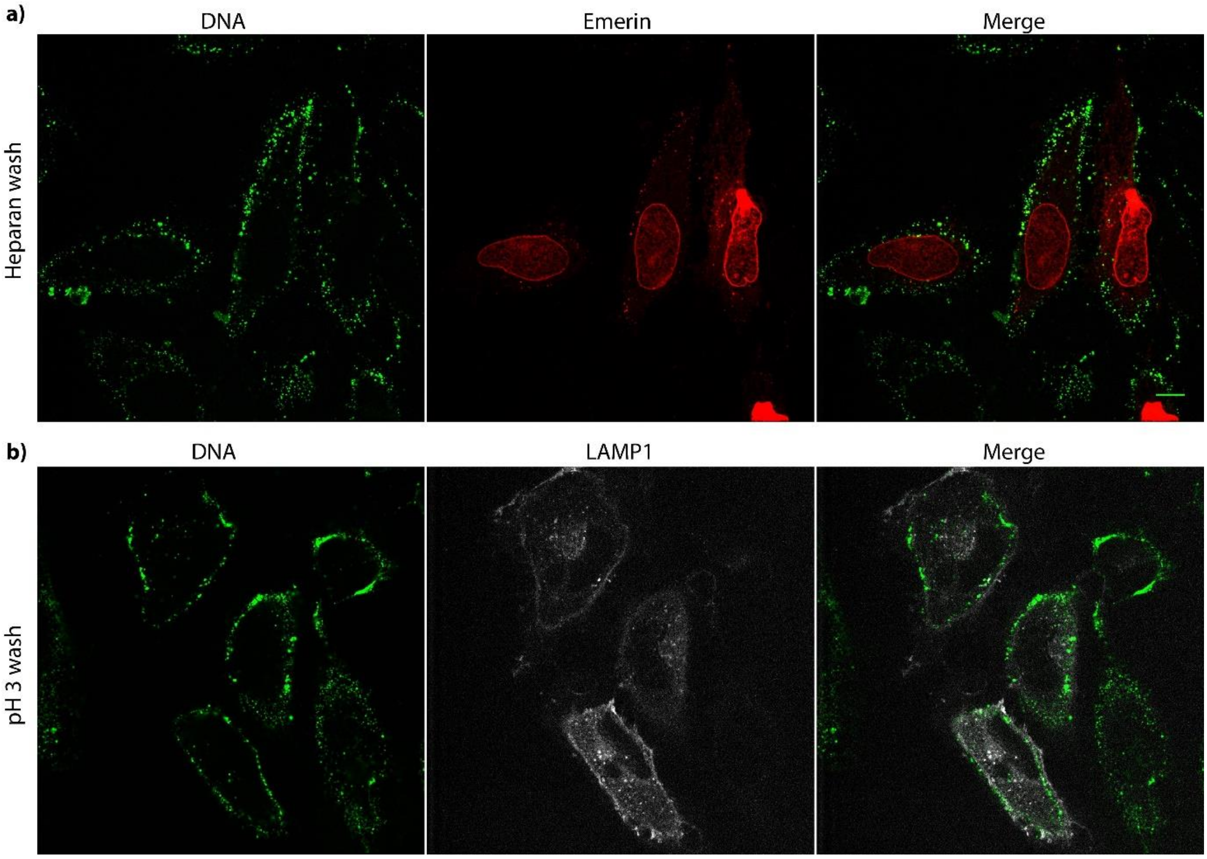

Figure S13. Confocal microscopy images of HeLa cells transfected with TFAMoplexes. The cells were washed prior to fixation with 40 mg mL^{-1} heparan sulfate (a) or pH 3 glycine buffer (b) in an attempt to remove particles from the cell surface. Shown is a single z-slice in the channels fluorescently labelled DNA (green), emerin-EGFP (red, a), LAMP1-turquoise (white, b) and the composite images. Scale bar 10 μm.

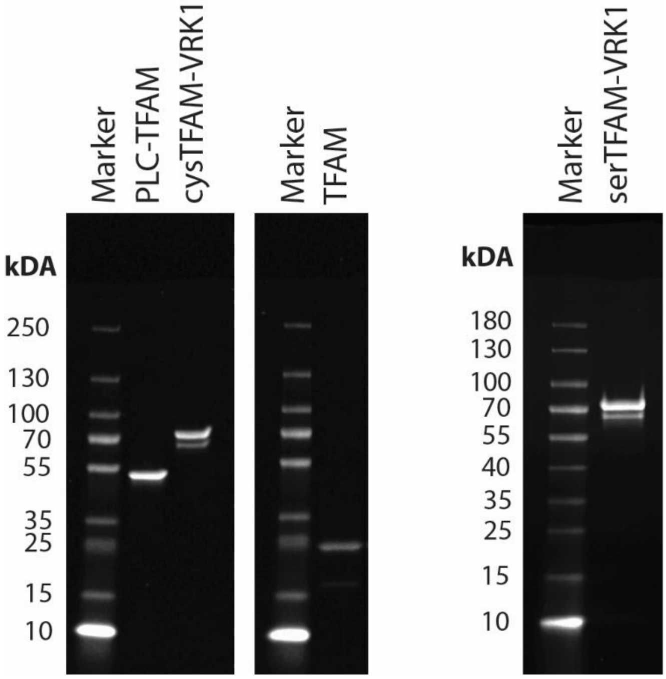

Figure S14. SDS-PAGE analysis of the main TFAM constructs. Shown are the final protein batches of PLC-TFAM, cysTFAM-VRK1, TFAM and serTFAM-VRK1. 1 µg protein was loaded per well. The gels were stained with Coomassie. Note: TFAM-VRK1 constructs generally contained impurities at 68-69 kDa (determined by LC-MS), which could not be removed completely.

Table S1. Summary TFAMoplex and LP3000 transfection of HeLa cells. Shown are the mean percentages of GFP positive cells (± SD). All transfections were performed with 200 ng DNA per mL cell medium for 30 min (if not stated otherwise). ND = Not determined.

		Transfection conditions			
		DMEM w/ 10% FBS		≥ 95% FBS	
Particle formation		TFAMoplex	LP3000	TFAMoplex	LP3000
	PBS/ OptiMEM	31.9 ± 1.1%	40.9 ± 4.0%[1]	8.7 ± 0.75%	9.9 ± 1.7%
	80% FBS	ND	ND	29.3 ± 6.9%	1.4 ± 0.4%

[(1)] 2 h transfection experiment instead of 30 min

Table S2. GeneArt Gene Synthesis DNA strings. DNA inserts synthesized for construct cloning.

No.	Name	Sequence
1	cysTFAM (A105C, V109C)	CCCCTCTAGAAATAATTTTGTTTAACTTTAAGAAGGAGATATACCATGGGTAGCA<br>GCCATCATCATCATCACCACGGTTcgGGATCCATGTCATCTGTCTTGGCAAGTTGTC<br>CAAAGAAACCTGTAAGTTCTTACCTTCGATTTTCTAAAGAACAACTACCCATATTT<br>AAAGCTCAGAACCCAGATGCAAAAACTACAGAACTAATTAGAAGAATTGCCCAG<br>CGTTGGAGGGAACTTCCTGATTCAAAGAAAAAAATATATCAAGATGCTTATAGGtg<br>cGAGTGGCAatgtTATAAAGAAGAGATAAGCAGATTTAAAGAgCAGtTgACTCCAAGT<br>CAGATTATGTCTTTGGAAAAAGAAATCATGGACAAACATTTAAAAAGGAAAGCT<br>ATGACAAAAAAAAAAGAGTTAACACTGCTTGGAAAACCAAAAAGACCTCGTTCA<br>GCTTATAACGTTTATGTAGCTGAAAGATTCCAAGAAGCTAAGGGTGATTCACCGC<br>AGGAAAAGCTGAAGACTGTAAAGGAAAACTGGAAAAATCTGTCTGACTCTGAAA<br>AGGAATTATATATTCAGCATGCTAAAGAGGACGAAACTCGTTATCATAATGAAAT<br>GAAGTCTTGGGAAGAACAAATGATTGAAGTTGGACGAAAGGATCTTCTACGTCGC<br>ACAATAAAGAAACAACGAAAATATGGTGCTGAGGAGTGTTCCGGTGGATCAAGC<br>GGCCGCAGTTAACTCGAGTC
2	TFAM (A105C)	CCCCTCTAGAAATAATTTTGTTTAACTTTAAGAAGGAGATATACCATGGGTAGCA<br>GCCATCATCATCATCACCACGGTTcgGGATCCATGTCATCTGTCTTGGCAAGTTGTC<br>CAAAGAAACCTGTAAGTTCTTACCTTCGATTTTCTAAAGAACAACTACCCATATTT

		AAAGCTCAGAACCCAGATGCAAAAACTACAGAACTAATTAGAAGAATTGCCCAGCGTTGGAGGGAACTTCCTGATTCAAAGAAAAAAATATATCAAGATGCTTATAGGtgcGAGTGGCAagtgTATAAAGAAGAGATAAGCAGATTTAAAGAgCAGtTgACTCCAAGTCAGATTATGTCTTTGGAAAAAGAAATCATGGACAAACATTTAAAAAGGAAAGCTATGACAAAAAAAAAAGAGcTtACACTGCTTGGAAAACCAAAAAGACCTCGTTCAGCTTATAACGTTTATGTAGCTGAAAGATTCCAAGAAGCTAAGGGTGATTCACCGCAGGAAAAGCTGAAGACTGTAAAGGAAAACTGGAAAAATCTGTCTGACTCTGAAAAGGAATTATATATTCAGCATGCTAAAGAGGACGAAACTCGTTATCATAATGAAATGAAGTCTTGGGAAGAACAAATGATTGAAGTTGGACGAAAGGATCTTCTACGTCGCACAATAAAGAAACAACGAAAATATGGTGCTGAGGAGTGTtccggtggatcaagcggccgcagttaaCTCGAGtc
3	cysTFAM (A105C, V109C)	gTcAAGCTTcgGGATCCATGTCATCTGTCTTGGCAAGTTGTCCAAAGAAACCTGTAAGTTCTTACCTTCGATTTTCTAAAGAACAACTACCCATATTTAAAGCTCAGAACCCAGATGCAAAAACTACAGAACTAATTAGAAGAATTGCCCAGCGTTGGAGGGAACTTCCTGATTCAAAGAAAAAAATATATCAAGATGCTTATAGGtgcGAGTGGCAatgtTATAAAGAAGAGATAAGCAGATTTAAAGAgCAGtTgACTCCAAGTCAGATTATGTCTTTGGAAAAAGAAATCATGGACAAACATTTAAAAAGGAAAGCTATGACAAAAAAAAAAGAGcTtACACTGCTTGGAAAACCAAAAAGACCTCGTTCAGCTTATAACGTTTATGTAGCTGAAAGATTCCAAGAAGCTAAGGGTGATTCACCGCAGGAAAAGCTGAAGACTGTAAAGGAAAACTGGAAAAATCTGTCTGACTCTGAAAAGGAATTATATATTCAGCATGCTAAAGAGGACGAAACTCGTTATCATAATGAAATGAAGTCTTGGGAAGAACAAATGATTGAAGTTGGACGAAAGGATCTTCTACGTCGCACAATAAAGAAACAACGAAAATATGGTGCTGAGGAGTGTtaaCTCGAGtc
4	PLC	ctactgcagGCTTCAAGCGAAAATCTGTATTTCCAATGGAGCGCGGACAATCCGACAAACACTGATGTAAACACCCATTATTGGCTTTTCAAGCAGGCTGAGAAAATTTTAGCAAAAGACGTGAATCATATGCGCGCAAACCTGATGAACGAACTTAAGAAGTTTGATAAACAAATAGCCCAGGGAATTTACGATGCCGACCACAAAAACCCCTATTATGACACTAGCACATTTCTGTCCCACTTCTATAACCCAGATCGCGATAACACGTACTTGCCTGGATTCGCAAATGCAAAGATTACGGGCGCTAAATATTTCAATCAAAGTGTTACGGACTATCGAGAAGGTAAATTCGATACAGCCTTCTACAAATTAGGTCTGGCCATCCACTATTACACAGATATTAGTCAACCTATGCACGCGAATAACTTCACGGCAATTAGCTATCCTCCGGGATATCATTGTGCATACGAAAACTACGTTGATACCATTAAACACAACTACCAGGCTACAGAAGATATGGTTGCCAAACGGTTTTGCAGCGACGACGTTA

		AAGATTGGTTATATGAAAACGCCAAACGTGCTAAAGCAGATTATCCGAAGATAGT AAACGCGAAAACAAAAAAAAGCTACCTCGTGGGAAACTCTGAATGGAAAAAGGA TACGGTCGAACCTACAGGAGCTCGCTTACGTGATTCACAGCAGACGTTAGCCGGG TTCTTAGAATTTTGGTCTAAAAAGACAAATGAAAGCGGTGGTTCTGGTGGATCGG GATCCAT
5	TFAM (V109C)	CCCCTCTAGAAATAATTTTGTTTAACTTTAAGAAGGAGATATACCATGGGTAGCA GCCATCATCATCATCACCACGGTTcgGGATCCATGTCATCTGTCTTGGCAAGTTGTC CAAAGAAACCTGTAAGTTCTTACCTTCGATTTTCTAAAGAACAACTACCCATATTT AAAGCTCAGAACCCAGATGCAAAAACTACAGAACTAATTAGAAGAATTGCCCAG CGTTGGAGGGAACTTCCTGATTCAAAGAAAAAAATATATCAAGATGCTTATAGGg cgGAGTGGCAatgtTATAAAGAAGAGATAAGCAGATTTAAAGAgCAGtTgACTCCAAG TCAGATTATGTCTTTGGAAAAAGAAATCATGGACAAACATTTAAAAAGGAAAGCT ATGACAAAAAAAAAAGAGTTAACACTGCTTGGAAAACCAAAAAGACCTCGTTCA GCTTATAACGTTTATGTAGCTGAAAGATTCCAAGAAGCTAAGGGTGATTCACCGC AGGAAAAGCTGAAGACTGTAAAGGAAAACTGGAAAAATCTGTCTGACTCTGAAA AGGAATTATATATTCAGCATGCTAAAGAGGACGAAACTCGTTATCATAATGAAAT GAAGTCTTGGGAAGAACAAATGATTGAAGTTGGACGAAAGGATCTTCTACGTCGC ACAATAAAGAAACAACGAAAATATGGTGCTGAGGAGTGTtccggtggatcaagcggccgcagt taaCTCGAGtc
6	TFAM (A105S, V109S)	CCCCTCTAGAAATAATTTTGTTTAACTTTAAGAAGGAGATATACCATGGGTAGCA GCCATCATCATCATCACCACGGTTcgGGATCCATGTCATCTGTCTTGGCAAGTTGTC CAAAGAAACCTGTAAGTTCTTACCTTCGATTTTCTAAAGAACAACTACCCATATTT AAAGCTCAGAACCCAGATGCAAAAACTACAGAACTAATTAGAAGAATTGCCCAG CGTTGGAGGGAACTTCCTGATTCAAAGAAAAAAATATATCAAGATGCTTATAGGtc tGAGTGGCAaagcTATAAAGAAGAGATAAGCAGATTTAAAGAgCAGtTgACTCCAAG TCAGATTATGTCTTTGGAAAAAGAAATCATGGACAAACATTTAAAAAGGAAAGCT ATGACAAAAAAAAAAGAGTTAACACTGCTTGGAAAACCAAAAAGACCTCGTTCA GCTTATAACGTTTATGTAGCTGAAAGATTCCAAGAAGCTAAGGGTGATTCACCGC AGGAAAAGCTGAAGACTGTAAAGGAAAACTGGAAAAATCTGTCTGACTCTGAAA AGGAATTATATATTCAGCATGCTAAAGAGGACGAAACTCGTTATCATAATGAAAT GAAGTCTTGGGAAGAACAAATGATTGAAGTTGGACGAAAGGATCTTCTACGTCGC ACAATAAAGAAACAACGAAAATATGGTGCTGAGGAGTGTtccggtggatcaagcggccgcagt taaCTCGAGtc

Table S3. Physiochemical parameters of the TFAM protein constructs. Calculated with ProtParam (ExPASy).

Number	Construct	Molar mass (Da)	Extinction coefficient ($M^{-1}cm^{-1}$)
1	His_6-TFAM-VRK1	73,202	98,210
2	His_6-cysTFAM-VRK1	73,241	98,210
3	TFAM(A105C)-VRK1	73,237	98,210
4	TFAM	26,005	35,410
5	cysTFAM	26,041	35,410
6	MBP- PLC-TFAM	97,618	159,060
7	PLC-TFAM	52,857	91,220
8	PLC-cysTFAM	52,893	91,220
9	TFAM-deadVRK1	73,147	98,210
10	cysTFAM-deadVRK1	73,197	98,210
11	cysTFAM-NLS	27,192	35,410
12	TFAM(V109C)-VRK1	73,209	98,210
13	TFAM(A105S, V109S)-VRK1	73,209	98,210
